# Antibacterial and anticancer activities of three novel lectin-conjugated chitosan nanoparticles

**DOI:** 10.1007/s00253-024-13344-7

**Published:** 2024-11-27

**Authors:** Mervat Mounir Soliman, Einas Hamed El-Shatoury, Magda Mahmoud Ibrahim El-Araby

**Affiliations:** 1 Department of Microbiology, Faculty of Science, Ain Shams University, Cairo, Egypt; 2 Department of Botany, Faculty of Science, Ain Shams University, Cairo, Egypt

**Keywords:** Antibacterial activities, Anticancer activities, Lectins, Chitosan nanoparticles

## Abstract

**Abstract:**

To the best of our knowledge, this is the first attempt to synthesize, characterize, and determine the antibacterial and anticancer effects of three novel conjugates of plant lectins: phytohemagglutinin lectin (PHA), soybean agglutinin (SBA), and peanut agglutinin (PNA) with chitosan nanoparticles (CHNPs). The lectin concentration within prepared conjugates was estimated using nannodrop, and the highest concentration was 0.96 mg/ml in PHA-CHNPs. SDS-PAGE showed the molecular weights of conjugates ranged from 26.9 to 63.9 kDa. UV spectrophotometer recorded the absorbance peaks of conjugates somewhere between 200 and 230 nm. Hemagglutination analysis verified the presence of actively binding lectins. The three conjugates showed strong antibacterial activity against Gram-positive and Gram-negative bacteria compared to pure lectins and chitosan nanoparticles. The highest inhibition zone was 55.67 ± 4.04, 38.67 ± 5.51, and 37.33 ± 2.52 for PHA-CHNPs against *Enterococcus faecalis*, *Salmonella typhimurium*, and *Shigella sonnei*, respectively, followed by 36.3 ± 0.15 for PNA-CHNPs against *Staphylococcus aureus*. The lowest MIC was 1.5 µg/ml for PHA-CHNPs against *Enterococcus faecalis*, followed by 12 µg/ml for PNA-CHNPs and SBA-CHNPs against *Salmonella typhimurium* and *Enterococcus faecalis*, respectively. TEM microphotographs show the conjugation pattern between lectins and chitosan nanoparticles and the morphological differences between control, treated bacteria, and cancer cells. Moreover, 100 μg/ml of PHA-CHNPs affect tongue carcinoma (HNO-97), colorectal cancer (HT-29), and human melanoma (A375) cancer cell lines, reducing cell viability by 38.78 ± 1.85%, 49.88 ± 1.11%, and 66.92 ± 3.60%, respectively. This study develops three innovative conjugates of lectin chitosan nanoparticles that need to be tested as potential antibacterial and anticancer agents for medical and cancer therapy applications.

**Key points:**

• *Lectin-conjugated chitosan nanoparticles exhibit antibacterial activity.*

• *All conjugates are safe for oral epithelial cells and human skin fibroblasts.*

• *The PHA-CHNP conjugates have anticancer activity against HNO-97, HT-29, and A375.*

**Supplementary Information:**

The online version contains supplementary material available at 10.1007/s00253-024-13344-7.

## Introduction

Cancer represents a significant threat to human health worldwide. By 2030, global cancer deaths are estimated to exceed 12 million (Fu et al. 2016). Cancer diseases occur because of the pathophysiological alterations in the normal process of cell division (Hanahan 2022). Moreover, the world is facing antibiotic resistance which arises when bacteria defeat the drugs, through different mechanisms, leading to an elevated risk of diseases and death (Levy and Marshall 2004; Maxson and Mitchell 2016). The above circumstances have generated an essential need to develop new drugs and efficient biomaterials to control or treat infectious and cancer diseases (Mohamed et al. 2020; Peng et al. 2022).

Natural biomaterials possess biodegradability, abundance, and renewability and lack toxicity. For example, chitosan nanoparticles are bioactive and ecofriendly materials that possess unique physical and chemical properties (Aliasghari et al. 2016). Furthermore, plant lectins are naturally occurring bioactive compounds that have been extracted and characterized from different parts of the plant. They are stable over a range of circumstances, including temperature and pH (Awadallah et al. 2017).

Plant lectins can inhibit bacterial growth through the interaction between their carbohydrate-binding proteins with bacterial membranes (Mishra et al. 2019). For example, lectins isolated from some legumes exhibited antibacterial properties against various pathogenic bacteria including *Bacillus* sp., *Escherichia coli*, and *Staphylococcus aureus* (Sammour and El-Shanshoury 1992; Hamed et al. 2017; El-Araby, et al. 2020). Moreover, chitosan nanoparticles have antibacterial activity which varies between Gram-negative and Gram-positive bacteria. Some previous studies recorded a potent antibacterial activity on Gram-positive bacteria compared to Gram-negative bacteria, but the other studies reported greater effectiveness against Gram-negative bacteria compared to Gram-positive bacteria (Chung et al. 2004).

Plant lectins can suppress cancer cells by initiating an immunological cascade that results in apoptosis or autophagy (Konozy and Osman 2022). For example, leukemia, sarcoma, hepatoma, and breast cancer have been effectively treated with plant lectins (Huldani et al. 2022). Their cytotoxicity against cancer is restricted to unmodified cells because they can distinguish between healthy and malignant cells (Reis et al. 2010). Moreover, the modification of nanoparticles with plant lectins can improve anticancer drug delivery through increasing intracellular delivery and targetability (De Oliveira Figueiroa et al. 2017).

In this research, lectin-conjugated chitosan nanoparticles were synthesized and characterized using different methods. Moreover, their antibacterial activity against human pathogenic bacteria and their cytotoxicity against normal and cancer cell lines were examined.

## Materials and methods

### Materials

#### Plant lectins

*Phaseolus vulgaris* agglutinin or phytohemagglutinin lectin (PHA), soybean agglutinin (SBA), and peanut agglutinin (PNA) were purchased from Sigma-Aldrich. Chitosan nanoparticles were purchased from Nanotech, Cairo, Egypt. Blood groups (types A, B, AB, and O) were obtained from healthy donors at a top lab medical laboratory in Cairo, Egypt.

#### Bacterial strains

*Enterococcus faecalis* ATCC 29212, *Staphylococcus aureus* ATCC 6538, *Escherichia coli* ATCC 25922, *Salmonella typhimurium* ATCC 14028, and *Shigella sonnei* ATCC 29930 were purchased from the Central Lab, Faculty of Science, Ain Shams University, Cairo, Egypt.

#### Cell lines

Normal cell lines (OEC (oral epithelial cell) and HSF (human skin fibroblast)) and cancer cell lines (HT-29, colorectal cancer; A375, human melanoma; and HNO-97, tongue carcinoma) were obtained from Nawah Scientific Center, Cairo, Egypt.

### Methods

#### Conjugation of lectins and chitosan nanoparticles

Plant lectins (PHA, PNA, SBA) are covalently conjugated to the surface of chitosan nanoparticles in a two-step method as previously described by Montisci et al. (2001) and Mishra et al. (2014), with slight modifications. The initial activation step for chitosan nanoparticles was as follows: 500 µl of chitosan nanoparticles (10 mg/ml) was washed using deionized water by centrifugation at 25,000 rpm for 10 min. The pellet was suspended in 500 µl of phosphate buffer saline (pH 7.4). Then, 0.25% glutaraldehyde (200, 300, and 400 µl) in water was added. The mixture was incubated at 30 °C for 3 h and was shaken gently to activate the hydroxyl groups.

The lectin conjugation to the activated chitosan nanoparticles was as follows: In brief, the suspension was rinsed four times in phosphate buffer saline (PBS 1X pH 7.4) and centrifuged at 10,000 rpm for 10 min to remove unreacted glutaraldehyde that may cross-link the lectin molecules. Then, 500 µl of phosphate buffer saline and 100 µl of lectin were added to the pellet, and the mixture was incubated overnight at room temperature. The mixture was centrifuged at 10,000 rpm for 10 min to remove free lectins and incubated for 1 h with 200 µl of ethanolamine (0.1 M) to block unreacted groups on the particles. The mixture was centrifuged, and the pellet was washed with phosphate buffer saline (pH 7.4) three times at 20,000 rpm for 20 min. Finally, the lectin chitosan nanoparticle conjugates were resuspended in 1 ml of phosphate buffer saline (pH 7.4) and kept at 2–8 °C.

#### Protein concentration and SDS-PAGE

The direct measurements of protein concentration within lectin-conjugated chitosan nanoparticles were performed at 280 nm using a microvolume spectrophotometer (Thermo Fisher Scientific, Nanodrop one/c microvolume UV–VIS spectrophotometer). Additionally, the molecular weight of lectins and lectin-conjugated chitosan nanoparticles (400 µl of 0.25% glutaraldehyde) was estimated, using the Laemmli SDS-PAGE with 12% separating gel and 5% stacking gel (Laemmli 1970).

#### UV–visible spectrophotometer (UV)

The absorption spectra of chitosan nanoparticles, lectins, and lectin-conjugated chitosan nanoparticles (400 µl of 0.25% glutaraldehyde) were measured using a UV–visible spectrophotometer with a wavelength range of 200 to 400 nm.

#### Hemagglutination activity (HA)

A hemagglutination activity test was used to check the presence of lectins. The assay was carried out in a 96-well plate using two-fold serial dilutions (1/8, 1/16, 1/32, 1/64, 1/128, 1/256). Each well has a mixture of 50 μl of phosphate buffer saline (pH 7.4), 50 μl of lectin-conjugated chitosan nanoparticles (PHA-CHNPs and SBA-CHNPs), and 50 μl of 4% human red blood cell types A, B, AB, and O (Wang et al. 2000). For PNA-CHNP conjugates, 50 μl of 4% human red blood cells were treated with 1 ml of neuraminidase (100 μg/ml). The mixture was gently mixed and incubated at 37 °C for an hour. Active lectins have the ability to agglutinate red blood cells by binding to the carbohydrates found on their surface, causing them to form a net shape. As a reference, a control well contained 50 μl of 4% red blood cell suspension and 50 μl of PBS, which gave a button shape inside the well (Wang et al. 2000).

#### The agar well diffusion method to test antibacterial activity

One hundred microliters of bacterial culture (10^6^ CFU/ml) was inoculated into agar plates containing Mueller–Hinton agar (MHA) at 37 °C for 24 h. Wells were filled with 200 μl of chitosan nanoparticles or lectin-conjugated chitosan nanoparticles, and 100 μl of 50 mM phosphate buffer solution (pH 7.4) was used as a control. The plates were placed in the refrigerator overnight for diffusion. The plates were then incubated at 37 °C for 24 h. The average inhibition zone diameters (mm) were measured to detect antimicrobial activity (Athanassiadis et al. 2009).

#### The minimum inhibitory concentration (MIC)

MIC was performed by the microbroth dilution method using inocula of 10^6^ CFU/ml for tested bacteria. Chitosan nanoparticles at a concentration of 10 mg/ml (CHNPs) and their conjugates using 400 μl of 0.25% glutaraldehyde (PHA-CHNPs, PNA-CHNPs, and SBA-CHNPs) were serially two-fold diluted to obtain 10 concentrations from 96 to 0.1875 μg/ml in 1 ml of Mueller–Hinton broth (MHB). Then, 10 μl of tested bacteria was added to each well. The growth control well contains inoculated broth without samples, and the negative control well contains only the broth. As positive controls, ciprofloxacin and gentamicin were analyzed for their Gram-positive and Gram-negative, respectively. After incubation for 24 h, optical density was measured at 400 nm using the microplate reader FLUOSTAR Omega. The minimum inhibitory concentration was determined to be the lowest concentration of the tested conjugates that prevented the growth of bacteria (Ellof 1998).

#### Transmission electron microscopy (TEM)

Transmission electron microscopy (TEM) was performed using a JEOL electron microscope (TEM 1200X) in the Central Lab, Faculty of Science, Ain Shams University, Cairo, Egypt. To determine the distribution and particle size of the chitosan nanoparticles, pure lectins (PHA, PNA, and SBA) and lectin-conjugated chitosan nanoparticles (PHA-CHNPs, PNA-CHNPs, and SBA-CHNPs) were prepared with a volume of 400 ml of 0.25% glutaraldehyde. Moreover, the tested bacteria were examined by TEM before and after treatment with lectin-conjugated chitosan nanoparticles (400 µl of 0.25% glutaraldehyde).

#### Cytotoxicity test

A cytotoxicity test was performed using sulforhodamine B (SRB) to determine cell viability. One hundred microliters of 5 × 10^3^ tested cell suspension (normal cell lines, OEC and HSF; cancer cell lines, HT-29, A375, and HNO-97) was placed in 96-well plates. 100 μl of the tested samples at two concentrations (1 and 10 µg/ml) was obtained for quick screening cytotoxicity against normal cell lines and at different concentrations (1, 3, 10, 30, and 100 µg/ml) to determine the cytotoxicity and IC_50_ against cancer cell lines. Then, the cells were immobilized by replacing the media with 150 μl of 10% trichloroacetic acid (TCA) and incubated at 4 °C for 1 h. The TCA solution was removed, and the cells were rinsed five times with distilled water. Seventy microliters of aliquots of a 0.4% w/v solution of SRB was added and incubated in a dark location at room temperature for 10 min. The plates were rinsed three times with a solution of 1% acetic acid and let to dry naturally overnight. One hundred fifty microliters of TRIS solution (10 mM) was used to dissolve the SRB stain that was attached to the protein. The absorbance at wavelengths of 540 nm was measured using an Infinite F50 microplate reader from Technische Analysegerate (TECAN), Switzerland (Skehan et al. 1990).

#### Statistical analysis

The statistical analysis was performed using the ANOVA (one-way analysis of variance) test (Armstrong et al. 2000) using SPSS (Statistical Package for Social Sciences) version 20 and GraphPad Prism 8. The data are shown as mean ± standard deviation (SD), and statistically significant values were defined as *p*-values less than 0.05.

## Results

### Lectins were efficiently conjugated to CHNPs

PHA, PNA, and SBA lectins were covalently conjugated to the surface of chitosan nanoparticles using three volumes of 0.25% glutaraldehyde (200, 300, and 400 µl). The highest concentration of protein, which resulted from the conjugation of three pure lectins and chitosan nanoparticles, was obtained when using 400 µl of 0.25% glutaraldehyde (Table S1). Therefore, 400 µl of 0.25% glutaraldehyde of the conjugates PHA-CHNPs, PNA-CHNPs, and SBA-CHNPs was used to perform most of the analysis in this study.

**Fig. 1:**
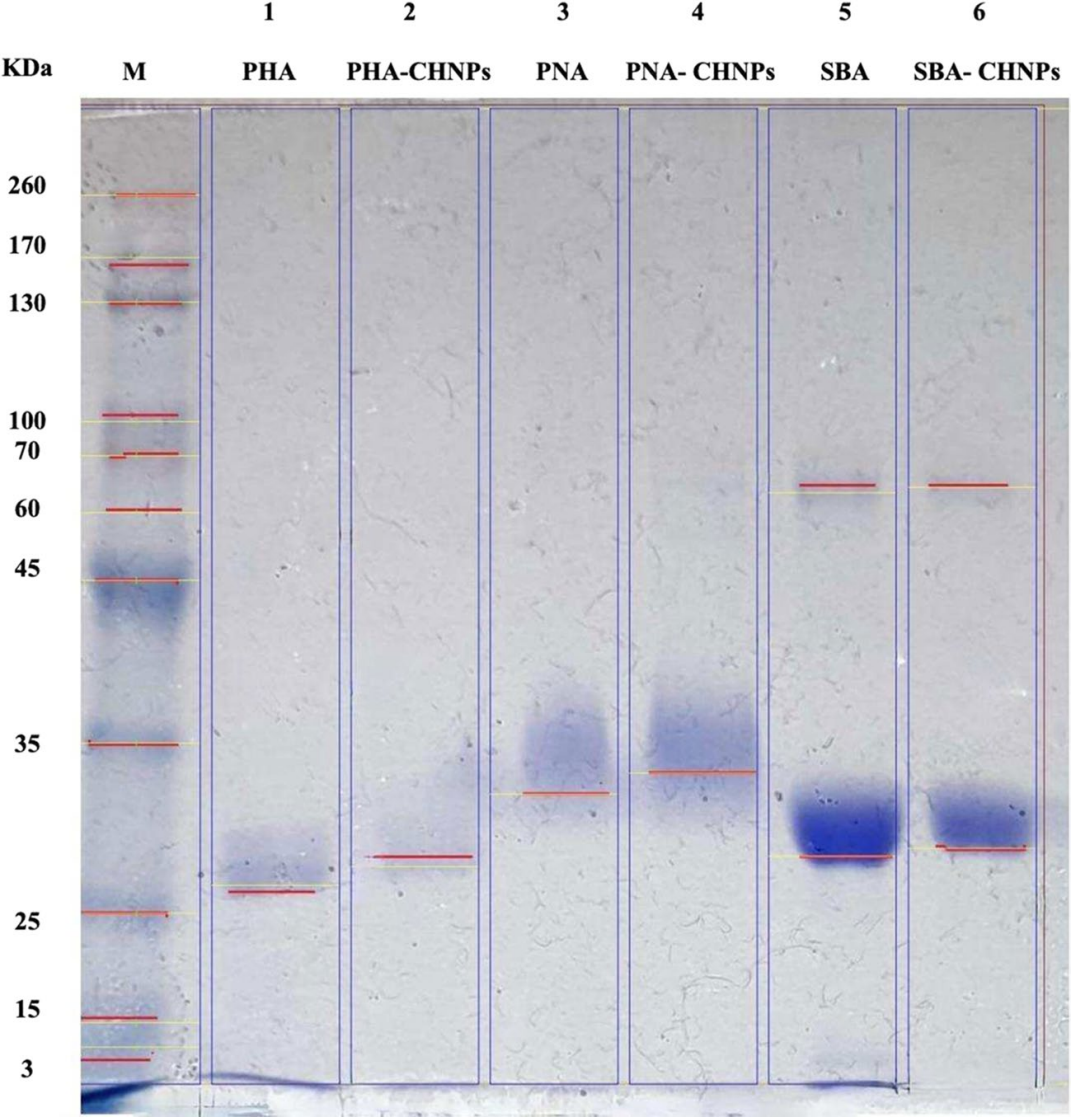
SDS-PAGE profile of M = marker; 1, 3, 5 = pure lectins PHA, PNA, and SBA, respectively; and 2, 4, 6 = lectin-conjugated chitosan nanoparticles PHA-CHNPs, PNA-CHNPs, and SBA-CHNPs, respectively, using 400 µl of 0.25% glutaraldehyde

SDS-PAGE analysis shows the molecular weight of lectin conjugated chitosan nanoparticles which was approximately like their pure lectins. SDS-PAGE gives one band for pure lectins PHA and PNA of molecular weight 26.9 and 32 kDa, respectively. Also, it shows two bands for SBA lectin with molecular weights of 27.9 and 63.8 kDa. Moreover, the molecular weight values for PHA-CHNPs and PNA-CHNPs were 27 and 31.6 kDa, respectively. In the case of SBA-CHNPs, SDS-PAGE gives two bands at the molecular weight 27.9 and 63.9 kDa (Fig. 1).

### UV–visible spectrophotometer (UV)

**Fig. 2:**
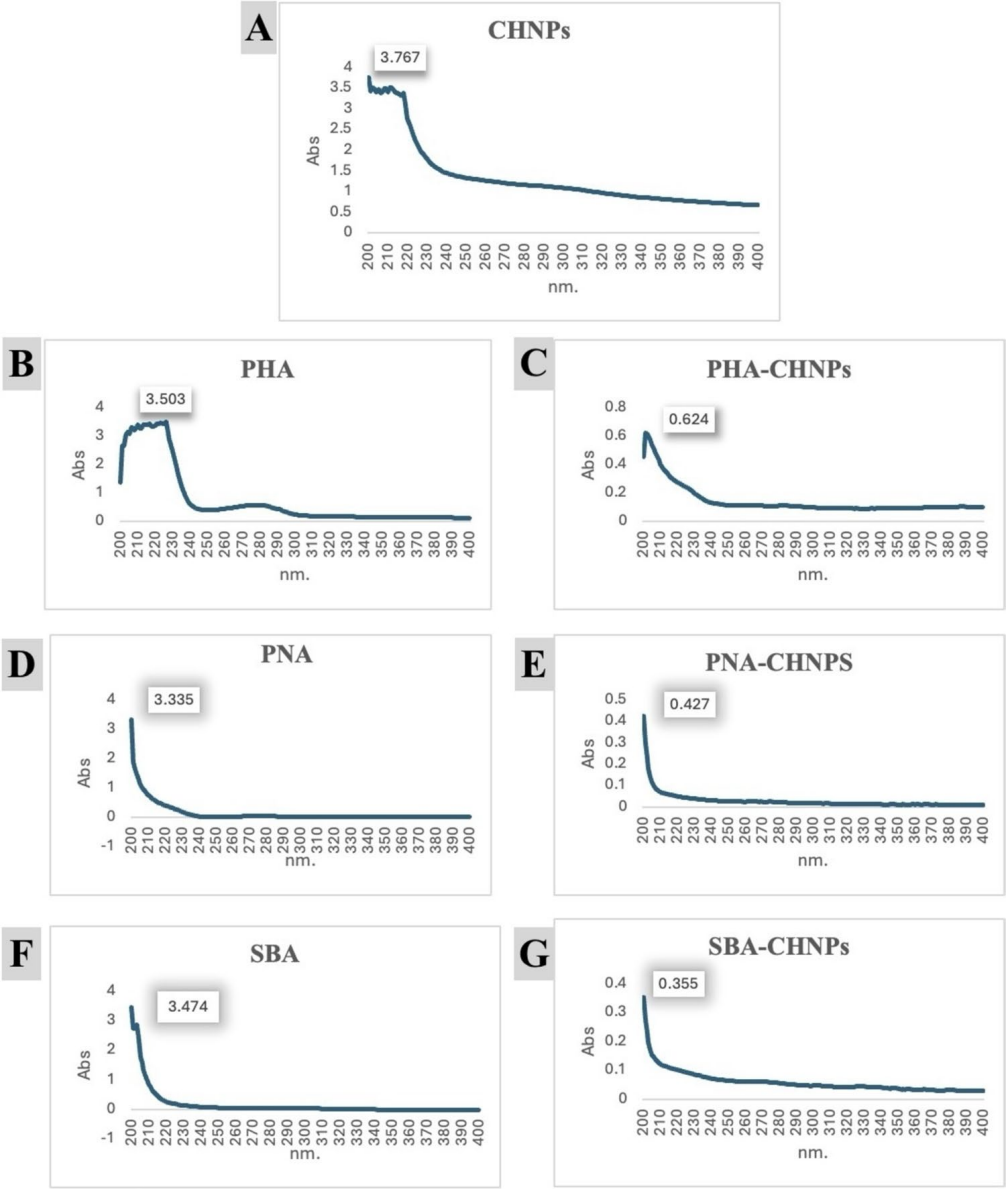
UV spectrophotometer absorbances of **A** chitosan nanoparticles (CHNPs); **B**, **D**, **F** pure lectins PHA, PNA, and SBA, respectively; and **C**, **E**, **G** lectin-conjugated chitosan nanoparticles PHA-CHNPs, PNA-CHNPs, and SBA-CHNPs, respectively, using 400 µl of 0.25% glutaraldehyde. PHA = phytohaemagglutinin lectin, PNA = peanut agglutinin lectin, and SBA = soybean agglutinin lectin

UV–visible scanning spectrophotometer was used to measure the absorbance of chitosan nanoparticles, lectins (PHA, PNA, and SBA), and lectin-conjugated chitosan nanoparticles at a wavelength range between 200 and 400 nm. The absorbance of chitosan nanoparticles was 3.767 at a wavelength of 200 nm. Moreover, the absorbance of PHA, PNA, and SBA lectins was 3.503, 3.335, and 3.474, respectively. However, after the conjugation, the absorbance at the same range of wavelengths for PHA-CHNPs, PNA-CHNPs, and SBA-CHNPs decreased to 0.624, 0.427, and 0.355, respectively (Fig. 2).

### Hemagglutination activity (HA)

All types of human blood groups were agglutinated by lectin-conjugated chitosan nanoparticles. Table S2 shows specific haemagglutination activities of conjugates which are expressed as titres. The results recorded that PHA-CHNPs gave the highest haemagglutination activity (256) for blood groups A and O. Furthermore, SBA-CHNPs and PNA-CHNPs recorded 64 and 16 for blood group AB, respectively.

### Antibacterial activity using agar well diffusion method

Table 1 shows the antibacterial activity of chitosan nanoparticles using three different concentrations (2.5, 5, and 10 mg/ml) and the antibacterial activity of three conjugates (PHA-CHNPs, PNA-CHNPs, and SBA-CHNPs) using three different volumes 200, 300, and 400 µl of 0.25% glutaraldehyde against five tested strains of Gram-positive and Gram-negative bacteria. The highest values were recorded at concentrations of 10 mg/ml for chitosan nanoparticles and for the three conjugates (PHA-CHNPs, PNA-CHNPs, and SBA-CHNPs) when using 400 µl of 0.25% glutaraldehyde. For Gram-negative bacteria, *Escherichia coli* showed the highest inhibition zone when treated with SBA-CHNPs (33 ± 3.61 mm), followed by PNA-CHNPs (24.7 ± 0.06 mm), PHA-CHNPs (15.33 ± 0.58 mm), and CHNPs (13.33 ± 1.53 mm). The *p*-value for this strain was less than 0.0001, indicating significant differences in antibacterial activity among the CHNPs. Similarly, for *Salmonella typhimurium*, PHA-CHNPs demonstrated the highest activity with an inhibition zone of 38.67 ± 5.51 mm, while PNA-CHNPs and SBA-CHNPs followed closely with inhibition zones of 35.98 ± 0.28 mm and 35.33 ± 0.58 mm, respectively. CHNPs exhibited the lowest activity (23.33 ± 1.53 mm), with a *p*-value of 0.003, confirming significant variations in efficacy. In the case of *Shigella sonnei*, PHA-CHNPs again showed the highest antibacterial activity (37.33 ± 2.52 mm), followed by PNA-CHNPs (34 ± 0.95 mm), SBA-CHNPs (24 ± 1 mm), and CHNPs (13 ± 2 mm). The *p*-value for this strain was less than 0.0001, indicating strong statistical significance (Table 1 and Fig. S1).

**Table 1:** Antibacterial activity of chitosan nanoparticles (CHNPs) using three different concentrations (2.5, 5, and 10 mg/ml) and three lectin-conjugated chitosan nanoparticles (PHA-CHNPs, PNA-CHNPs, and SBA-CHNPs) using three volumes (200, 300, 400 µl) of 0.25% glutaraldehyde against five tested strains of bacteria. Results are expressed as mean ± standard deviation

Concentration of CHNPs (mg/ml)		Inhibition zone (mm)						
		*Escherichia coli* ( −)	*Salmonella typhimurium* ( −)	*Shigella sonnei* ( −)	*Enterococcus faecalis* ( +)	*Staphylococcus aureus* ( +)	*p-*value	Statistically significant
CHNPs	**2.5**	8 ± 1	11.67 ± 1.15	10 ± 1	13 ± 1	10 ± 1	0.0015	**Sig**
	**5**	10 ± 1	13.33 ± 1.53	14 ± 1	15.33 ± 0.58	12.67 ± 1.15	0.0017	**Sig**
	**10**	13.33 ± 1.53	23.33 ± 1.53	13 ± 2	24 ± 1	21 ± 1	< 0.0001	**Sig**
Conjugates with 0.25% glutaraldehyde (µl)								
PHA-CHNPs	**200**	8 ± 1	33.33 ± 5.77	23 ± 2.65	26 ± 3.61	11.33 ± 1.53	< 0.0001	**Sig**
	**300**	12.67 ± 2.08	33.67 ± 1.53	34 ± 1.73	52 ± 2.65	32.33 ± 1.53	< 0.0001	**Sig**
	**400**	15.33 ± 0.58	38.67 ± 5.51	37.33 ± 2.52	55.67 ± 4.04	33.33 ± 1.15	< 0.0001	**Sig**
PNA-CHNPs	**200**	13.33 ± 1.53	31.54 ± 0.27	24 ± 1.97	14.33 ± 1.15	19 ± 1	< 0.001	**Sig**
	**300**	20.33 ± 0.58	33.34 ± 0.51	34 ± 0.51	19.67 ± 0.58	23 ± 2	< 0.001	**Sig**
	**400**	24.7 ± 0.06	35.98 ± 0.28	34 ± 0.95	25 ± 0.1	36.3 ± 0.15	< 0.0001	**Sig**
SBA-CHNPs	**200**	12 ± 1	24.33 ± 0.58	16.33 ± 1.53	9.33 ± 1.15	20 ± 1	< 0.0001	**Sig**
	**300**	24.67 ± 0.58	30.33 ± 0.58	21 ± 1	14.33 ± 1.15	24.67 ± 0.58	< 0.0001	**Sig**
	**400**	33 ± 3.61	35.33 ± 0.58	24 ± 1	21.33 ± 1.53	34 ± 2.65	< 0.0001	**Sig**

( −) = Gram-negative bacteria, ( +) = Gram-positive bacteria, *p*-value ≤ 0.05 considered statistically significant (95% confidence interval)

*CHNPs* chitosan nanoparticles; *PHA-CHNPs* phytohemagglutinin lectin–conjugated chitosan nanoparticles; *PNA-CHNPs* peanut agglutinin lectin–conjugated chitosan nanoparticles; *SBA-CHNPs* soybean agglutinin lectin–conjugated chitosan nanoparticles; *NI* no inhibition zone, non-significant; *Sig.* significance

For Gram-positive bacteria, *Enterococcus faecalis* exhibited the highest inhibition zone when treated with PHA-CHNPs (55.67 ± 4.04 mm), which significantly outperformed PNA-CHNPs (25 ± 0.1 mm), CHNPs (24 ± 1 mm), and SBA-CHNPs (21.33 ± 1.53 mm). The *p*-value was less than 0.0001, reflecting substantial differences in antibacterial effectiveness. Similarly, for *Staphylococcus aureus*, PNA-CHNPs exhibited the highest inhibition zone (36.3 ± 0.15 mm), followed closely by SBA-CHNPs (34 ± 2.65 mm), PHA-CHNPs (33.33 ± 1.15 mm), and CHNPs (21 ± 1 mm). The *p*-value for this strain was also less than 0.0001, signifying significant differences in antibacterial activity (Table 1 and Fig. S1).

### The minimum inhibitory concentration (MIC)

Table 2 shows MIC values for chitosan nanoparticles and lectin-conjugated chitosan nanoparticles (PHA-CHNPs, PNA-CHNPs, and SBA-CHNPs) against tested Gram-positive and Gram-negative bacteria. The lowest MIC value for chitosan nanoparticles (CHNPs) was 12 µg/ml against *Salmonella typhimurium* followed by 24 µg/ml against *Enterococcus faecalis*, while the MIC value of chitosan nanoparticles for the other three tested bacteria was 48 µg/ml.

**Table 2:** Minimum inhibitory concentration (MIC) of chitosan nanoparticles (CHNPs), pure lectins (PHA, PNA, SBA), and their conjugates (PHA-CHNPs, PNA-CHNPs, and SBA-CHNPs) using 400 µl of 0.25% glutaraldehyde against five strains of bacteria

Tested bacteria	MIC (µg/ml)							MIC of standards (µg/ml)	
	CHNPs	PHA	PHA-CHNPs	PNA	PNA-CHNPs	SBA	SBA-CHNPs	Gentamicin	Ciprofloxacin
*Escherichia coli* ( −)	48	NI	48	NI	96	NI	48	1.5	1.5
*Salmonella typhimurium* ( −)	12	96	48	24	12	12	96	24	NI
*Shigella sonnei* ( −)	48	96	48	48	24	24	96	24	3
*Enterococcus faecalis* ( +)	24	48	1.5	12	48	24	12	6	6
*Staphylococcus aureus* ( +)	48	48	24	12	48	48	24	3	3

MIC = the lowest concentration of antibacterial substance, lectin-conjugated chitosan nanoparticles, that inhibits the growth of bacteria. ( −) = Gram-negative bacteria, ( +) = Gram-positive bacteria

*CHNPs* chitosan nanoparticles, *PHA* phytohemagglutinin lectin, *PNA* peanut agglutinin, *SBA* soybean agglutinin, *PHA-CHNPs* phytohemagglutinin lectin–conjugated chitosan nanoparticles, *PNA-CHNPs* peanut agglutinin lectin–conjugated chitosan nanoparticles, *SBA-CHNPs* soybean agglutinin lectin–conjugated chitosan nanoparticles, *NI* no inhibition

The lowest MIC was 1.5 and 12 µg/ml for conjugates PHA-CHNPs and SBA-CHNPs, respectively, against *Enterococcus faecalis*. Moreover, MIC for PNA-CHNPs was 12 µg/ml against *Salmonella typhimurium*. While the MIC test gave no inhibition for pure lectins (PHA, PNA, and SBA) against *Escherichia coli*, the inhibition values for their conjugates PHA-CHNPs, PNA-CHNPs, and SBA-CHNPs were 48, 96, and 84 µg/ml, respectively, against the same bacteria (Table 2).

### Transmission electron microscopy (TEM)

**Fig. 3:**
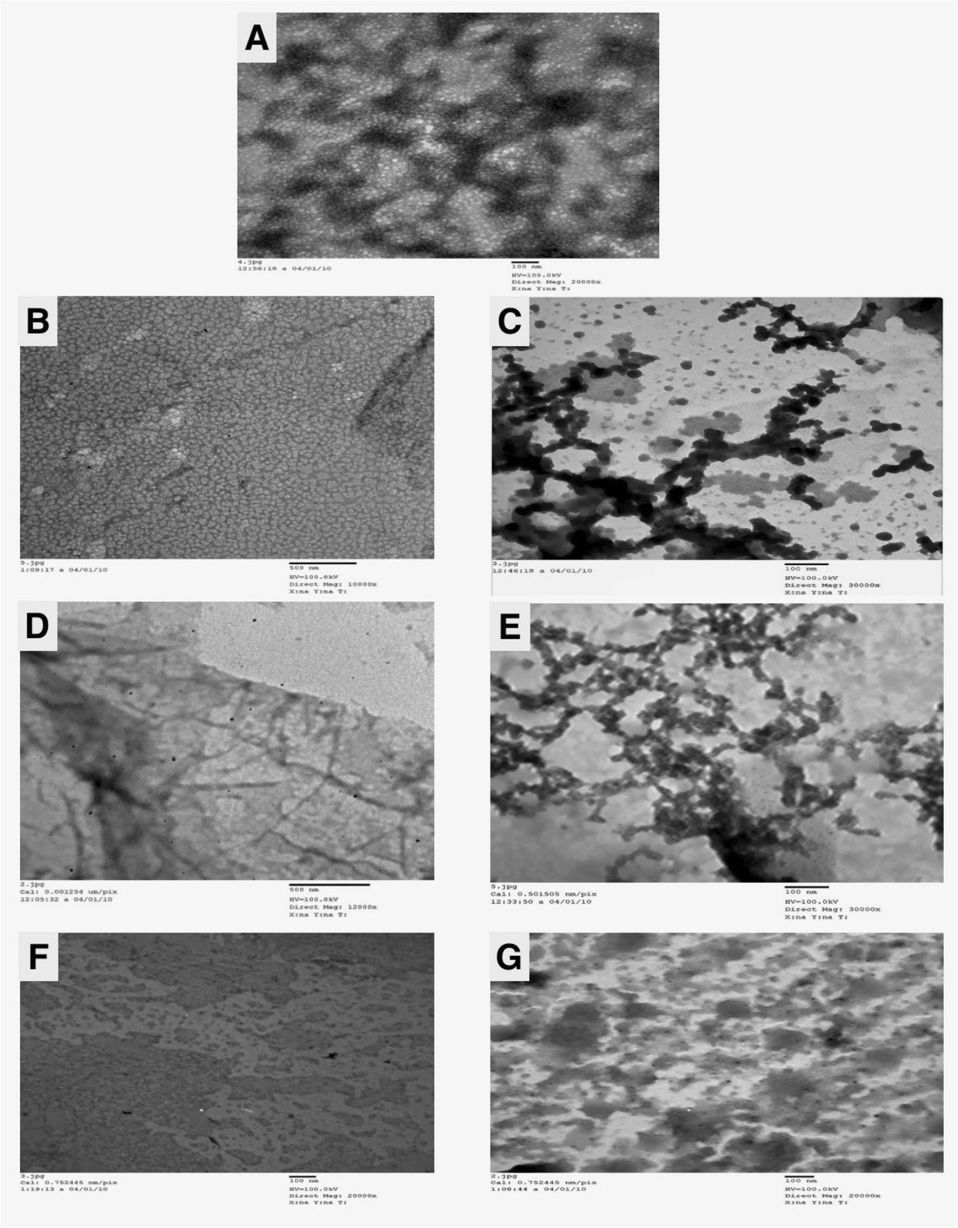
TEM photomicrographs of **A** chitosan nanoparticles; **B**, **D**, **F** pure lectins PHA, PNA, and SBA respectively; **C**, **E**, **G** lectin-conjugated chitosan nanoparticles PHA-CHNPs, PNA-CHNPs, and SBA-CHNPs, respectively, using 400 µl of 0.25% glutaraldehyde. PHA = phytohaemagglutinin lectin, PNA = peanut agglutinin lectin, and SBA = soybean agglutinin lectin

TEM microphotographs show chitosan nanoparticles with a size of 20 ± 5 nm. Photomicrographs also show the unique arrangement of pure plant lectins. The conjugation process, using 400 µl of 0.25% glutaraldehyde, significantly alters the distribution pattern of chitosan nanoparticles and pure lectins, leading to the formation of lectin-conjugated chitosan nanoparticles (PHA-CHNPs, PNA-CHNPs, and SBA-CHNPs) (Fig. 3). Furthermore, the microphotographs demonstrate an increase in the size of the conjugates that fall into the ranges of 30–35 nm, 25.2–28 nm, and 36.3–128 nm, respectively (Fig. S2, S3, and S4).

Lectin-conjugated chitosan nanoparticles are more efficient in inhibiting bacterial growth than chitosan nanoparticles or pure lectins. Moreover, TEM microphotographs show the tested Gram-positive and Gram-negative bacteria before and after treatment with chitosan nanoparticles (CHNPs), pure plant lectins (PHA, PNA, and SBA), and lectin-conjugated chitosan nanoparticles (PHA-CHNPs, PNA-CHNPs, and SBA-CHNPs) (Fig. S5, S6, S7, S8, and S9).

### Cytotoxicity analysis

Table 3 shows the results of quick screening cytotoxicity using sulforhodamine B (SRB) for chitosan nanoparticles (CHNPs) with a concentration of 10 mg/ml, pure lectins (PHA, PNA, and SBA), and their conjugates using 400 µl of 0.25% glutaraldehyde (PHA-CHNPs, PNA-CHNPs, and SBA-CHNPs) at two concentrations (10 and 100 μg/ml) against two normal cell lines (OEC and HSF). The results expressed in terms of cell viability percentages (mean ± SD) showed that all tested samples at concentrations of 10 and 100 μg/ml had no cytotoxic effect against oral epithelial cells (OEC) or human skin fibroblasts (HSF).

**Table 3:** Quick cytotoxicity screening using SRB for chitosan nanoparticles at concentrations of 10 mg/ml (CHNPs), pure lectins (PHA, PNA, and SBA), and their conjugates using 400 µl of 0.25% glutaraldehyde (PHA-CHNPs, PNA-CHNPs, and SBA-CHNPs) at two concentrations (10 and 100 μg/ml) against two normal cell lines (OEC and HSF). The results are expressed in terms of cell viability percentages as mean ± standard deviation

Normal cell lines	Cell viability (%)							
	Concentration of conjugates (µg/ml)	CHNPs	PHA	PHA-CH NPs	PNA	PNA-CHNPs	SBA	SBA-CHNPs
OEC: oral epithelial cell	10	99.17 ± 0.87	97.97 ± 0.45	97.25 ± 0.91	98.99 ± 1.32	98.16 ± 0.53	98.06 ± 1.45	90.91 ± 0.56
	100	99.79 ± 0.59	97.05 ± 0.98	96.56 ± 0.63	96.05 ± 1.51	96.72 ± 0.43	92.58 ± 1.92	97.58 ± 1.56
HSF: human skin fibroblast	10	98.59 ± 0.72	96.02 ± 0.87	96.23 ± 0.78	98.94 ± 1.96	102.67 ± 1.97	96.82 ± 1.74	99.32 ± 0.57
	100	97.67 ± 0.53	95.58 ± 0.22	96.37 ± 0.51	102.23 ± 2.84	101.86 ± 2.89	98.46 ± 1.23	97.16 ± 0.36

*CHNPs* chitosan nanoparticles, *PHA* phytohemagglutinin lectin, *PNA* peanut agglutinin, *SBA* soybean agglutinin, *PHA-CHNPs* phytohemagglutinin lectin–conjugated chitosan nanoparticles, *PNA-CHNPs* peanut agglutinin lectin–conjugated chitosan nanoparticles, *SBA-CHNPs* soybean agglutinin lectin–conjugated chitosan nanoparticles

**Fig. 4:**
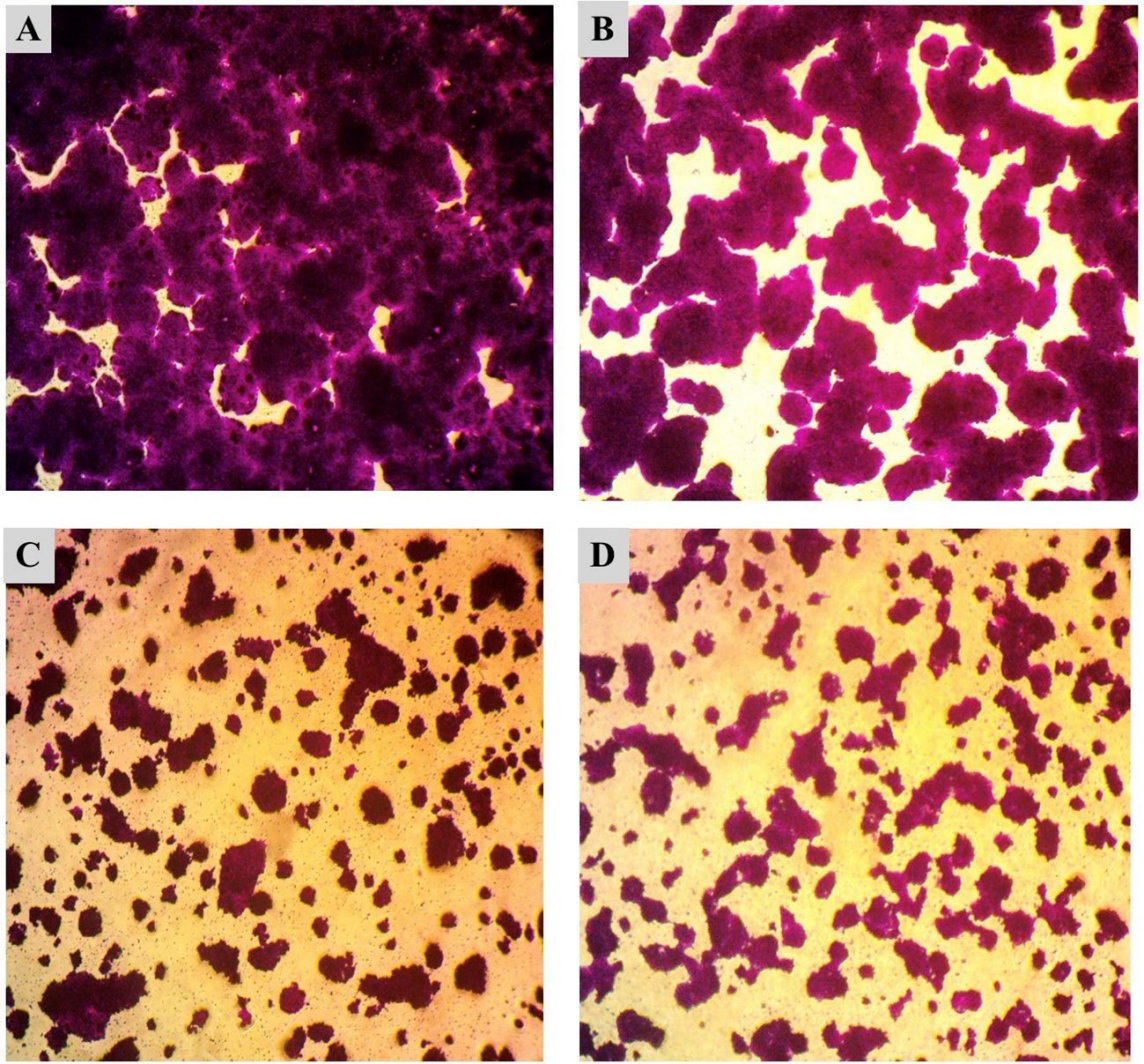
Microscopic photomicrographs of colorectal cancer (HT-29) that have been stained with SRB (sulforhodamine B). **A **Control cell lines, **B** chitosan nanoparticles (CHNPs), **C** phytohemagglutinin lectin (PHA), and **D** phytohemagglutinin lectin–conjugated chitosan nanoparticles (PHA-CHNPs) using 400 µl of 0.25% glutaraldehyde at concentration 100 μg/ml

**Fig. 5:**
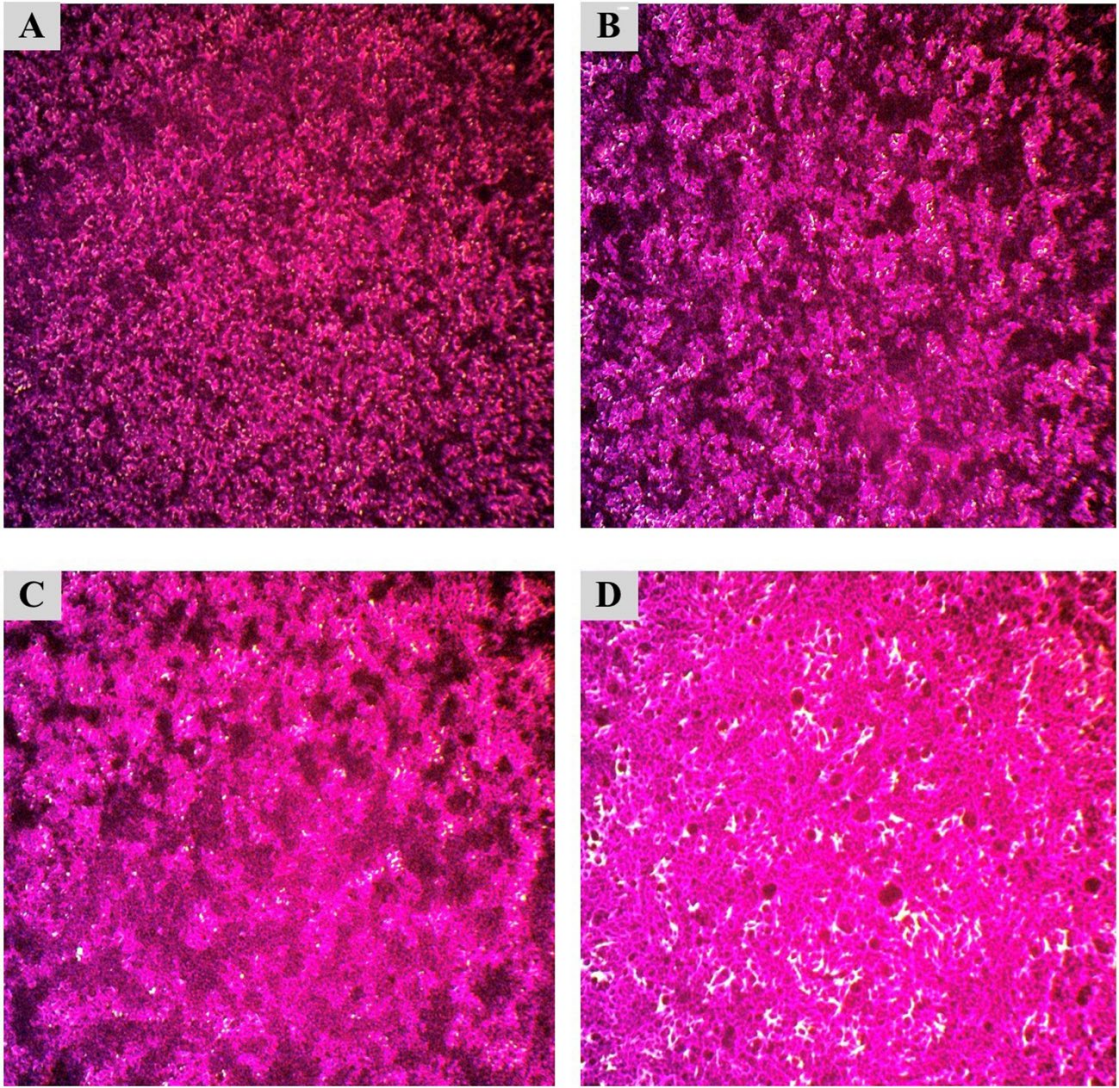
Microscopic photomicrographs of human melanoma (A375) that have been stained with SRB (sulforhodamine B). **A** Control cell lines, **B** chitosan nanoparticles (CHNPs), **C** phytohemagglutinin lectin (PHA), and **D** phytohemagglutinin lectin–conjugated chitosan nanoparticles (PHA-CHNPs) using 400 µl of 0.25% glutaraldehyde at concentration 100 μg/ml

**Fig. 6:**
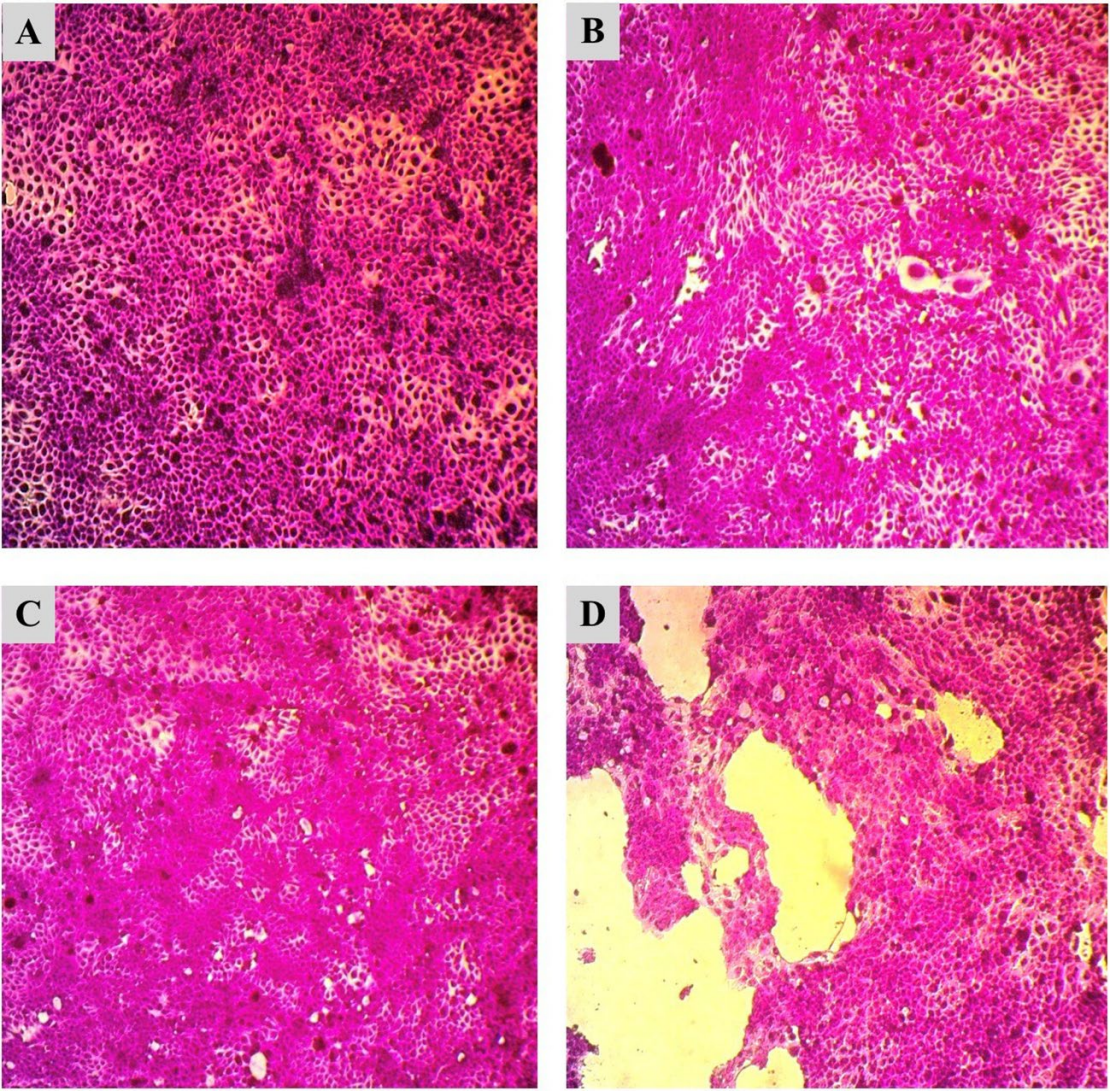
Microscopic photomicrographs of tongue carcinoma (HNO-97) that have been stained with SRB (sulforhodamine B). **A** Control cell lines, **B** chitosan nanoparticles (CHNPs), **C** phytohemagglutinin lectin (PHA), and **D** phytohemagglutinin lectin–conjugated chitosan nanoparticles (PHA-CHNPs) using 400 µl of 0.25% glutaraldehyde at concentration 100 μg/ml

Moreover, Table 4 shows the percentage of cell viability and IC_50_ values as results of cytotoxicity tests of chitosan nanoparticles (CHNPs), pure lectins (PHA, PNA, and SBA), and their conjugates (PHA-CHNPs, PNA-CHNPs, and SBA-CHNPs) at five concentrations (1, 3, 10, 30, and 100 μg/ml) on three cancer cell lines (HT-29, A375, and HNO-97). Figures 4, 5, and 6 show that the most sensitive cancer cell lines when treated with 100 μg/ml of PHA-CHNPs were tongue carcinoma (HNO-97), followed by colorectal cancer (HT-29), and human melanoma (A375), with cell viability percentages of 38.78 ± 1.85%, 49.88 ± 1.11%, and 66.92 ± 3.60%, respectively. Their corresponding IC_50_ values were 31.69, 88.68, and 135.58 μg/ml, respectively.

**Table 4:** The cytotoxicity of chitosan nanoparticles (CHNPs) at concentration 10 mg/ml (CHNPs), pure lectins (PHA, PNA, SBA), and their conjugates using 400 µl of 0.25% glutaraldehyde (PHA-CHNPs, PNA-CHNPs, and SBA-CHNPs) at five concentrations (1, 3, 5, 10, and 100 μg/ml) against three cancer cell lines (HT-29, A375, and HNO-97). The results are expressed in terms of cell viability percentages and IC_50_ values as mean ± standard deviation

Cancer cell lines	Conc. (µg/ml)	Cell viability (%)						
		CHNPs	PHA	PHA-CHNPs	PNA	PNA-CHNPs	SBA	SBA-CHNPs
HT-29: colorectal cancer	1	101.30 ± 0.32	99.50 ± 0.39	102.28 ± 0.16	100.76 ± 0.30	99.70 ± 0.40	100.85 ± 0.68	100.12 ± 1.44
	3	100.41 ± 1.07	99.68 ± 0.26	94.69 ± 2.39	100.69 ± 0.85	99.46 ± 0.15	100.09 ± 0.39	98.72 ± 0.72
	10	99.93 ± 0.25	98.06 ± 0.27	68.70 ± 0.22	99.68 ± 0.61	99.32 ± 0.39	99.94 ± 0.44	98.16 ± 0.38
	30	99.01 ± 1.22	83.32 ± 1.71	68.54 ± 0.62	99.63 ± 1.16	98.96 ± 1.52	98.32 ± 1.91	96.61 ± 3.14
	100	96.53 ± 2.70	57.93 ± 1.64	49.88 ± 1.11	99.47 ± 0.27	89.48 ± 1.61	97.43 ± 2.21	95.17 ± 3.07
IC_50_		> 100	128.53	88.68	> 100	> 100	> 100	> 100
A375: human melanoma	1	100.63 ± 0.16	102.64 ± 2.44	100.04 ± 0.15	100.25 ± 1.34	100.53 ± 0.53	100.61 ± 1.53	100.31 ± 0.15
	3	100.05 ± 0.15	100.28 ± 0.15	98.66 ± 2.19	99.67 ± 0.25	100.04 ± 0.15	99.79 ± 0.28	99.52 ± 0.15
	10	99.52 ± 0.29	99.69 ± 0.28	98.16 ± 1.57	98.60 ± 0.48	99.42 ± 0.38	98.91 ± 0.35	98.92 ± 0.13
	30	99.48 ± 0.24	98.61 ± 0.52	97.33 ± 2.53	98.45 ± 0.26	99.27 ± 0.14	98.86 ± 0.22	95.11 ± 3.33
	100	97.89 ± 0.12	97.01 ± 0.82	66.92 ± 3.60	97.33 ± 0.75	99.07 ± 3.97	98.17 ± 0.50	94.93 ± 3.60
IC_50_		> 100	> 100	135.58	> 100	> 100	> 100	> 100
HNO-97: tongue carcinoma	1	100.32 ± 1.84	101.19 ± 1.34	102 ± 1.16	100.20 ± 1.15	97.99 ± 0.75	98.67 ± 3.20	101.28 ± 2.22
	3	99.06 ± 1.71	100.16 ± 2.60	99.19 ± 2.23	99.65 ± 0.79	94.71 ± 0.68	96.14 ± 2.27	98.14 ± 2.90
	10	97.24 ± 1.48	97.76 ± 1.13	94.64 ± 1.46	99.43 ± 0.83	91.81 ± 0.78	91.68 ± 2.07	93.06 ± 2.02
	30	96.90 ± 0.18	94.08 ± 2.06	68.61 ± 2.71	88.90 ± 1.64	90.02 ± 3.16	91.42 ± 2.69	95.71 ± 2.45
	100	94.81 ± 0.85	91.92 ± 1.32	38.78 ± 1.85	71.41 ± 2.58	74.55 ± 2.69	88.45 ± 0.54	89.70 ± 1.69
IC_50_		> 100	> 100	31.69	221.54	601.09	> 100	> 100

Half the maximal inhibitory concentration (IC_50_) is the concentration of a medication or inhibitor required to inhibit a biological process or reaction by 50%

*CHNPs* chitosan nanoparticles, *PHA* phytohemagglutinin lectin, *PNA* peanut agglutinin, *SBA* soybean agglutinin, *PHA-CHNPs* phytohemagglutinin lectin–conjugated chitosan nanoparticles, *PNA-CHNPs* peanut agglutinin lectin–conjugated chitosan nanoparticles, *SBA-CHNPs* soybean agglutinin lectin–conjugated chitosan nanoparticles

Table 4 shows the cytotoxicity of pure lectin (PHA) on colorectal cancer (HT-29) with a cell viability percentage of 57.93 ± 1.64% and an IC_50_ 128.53 μg/ml, while the pure lectins of PNA and its conjugates with chitosan nanoparticles (PNA-CHNPs) at concentrations of 100 μg/ml have a low cytotoxic effect on tongue carcinoma (HNO-97) with cell viability percentages of 71.41 ± 2.58 and 74.55 ± 2.69, respectively, and their IC_50_ values were calculated theoretically to be 221.54 and 601.09 μg/ml, respectively. Furthermore, the microscope microphotographs that have been stained with sulforhodamine B (SRB) documented changes in cancer cell lines before and after treatment with chitosan nanoparticles, pure lectins, and conjugates at concentrations of 100 µg/ml (Figs. 4, 5, and 6).

## Discussion

In recent years, cancer has become a significant field of study worldwide due to the absence of a definitive treatment. In 2020, there were over 19.3 million documented and diagnosed cases of cancer, resulting in approximately 10 million deaths (Ferlay et al. 2021). Furthermore, the world is confronted with the issue of bacterial resistance, which diminishes the effectiveness of existing antibiotics and makes the treatment of infectious diseases more difficult or even impossible, leading to a rise in death rates (Chen et al. 2014).

Based on the previous circumstances, scientific research has shown that chitosan nanoparticles have promising potential as anticancer agents (Shakil et al. 2021) and antibacterial agents (Chandrasekaran et al. 2020). Moreover, plant lectins are effective tools for controlling and inhibiting bacterial diseases (Dias et al. 2015), and they function as anticancer agents with promising therapeutic capabilities (Huldani et al. 2022). The abundance of amino groups on the surface of chitosan nanoparticles makes them very appropriate for effective conjugation with plant lectin, since they promote fast coupling. Moreover, glutaraldehyde is a commonly used crosslinking agent used to link chitosan nanoparticles because of the hydroxyl groups found in their neighboring chains (Yu et al. 2011).

In this study, the conjugation process of plant lectins namely phytohemagglutinin lectin (PHA), peanut agglutinin (PNA), soybean agglutinin (SBA), and chitosan nanoparticles (CHNPs) follows a two-step procedure which is activation step and coupling step as previously described by Montisci et al. (2001), who used two other plant lectins, namely, *Lycopersicon esculentum* L. and *Lotus tetragonolobus* lectins that linked to tiny poly microspheres. Moreover, Gupta et al. (2006) conjugated peanut lectin (PNA) with polylactic co-glycolic acid (PLGA) nanoparticles.

To the best of our knowledge, for the first time, this study records effective conjugation between chitosan nanoparticles (CHNPs) and pure lectins (PHA, PNA, and SBA) using different volumes (200, 300, and 400 µl) of 0.25% of glutaraldehyde, and the highest protein concentration and the most effective conjugation were recorded when using 400 µl of 0.25% glutaraldehyde. These results are supported by the previous findings which recorded successful conjugation between *Lotus tetragonolobus* agglutinin (LTA) lectin with chitosan nanoparticles (CHNPs) using the same volumes 200, 300, and 400 µl of 0.25% glutaraldehyde, and the higher conjugation was recorded at volume 200 µl of glutaraldehyde (Mishra et al. 2014).

In this study, SDS-PAGE analysis revealed that both the pure lectins and lectin-conjugated chitosan nanoparticles showed bands with almost identical molecular weights. Pure lectin (PHA) and its conjugate with (PHA-CHNPs) produce a single band with 27 kDa. Pure lectin (SBA) and its conjugate (SBA-CHNPs) have the same molecular weight of 27.9 kDa.These results are consistent with Tsaneva and Van Damme (2020), who found that most seed lectins have a molecular weight ranging from 25 to 30 kDa. Moreover, this study found that pure lectin (SBA) and its conjugate (SBA-CHNPs) have a band with the same molecular weight of 64 kDa.

The previous study confirms that soybean lectin storage proteins consist of two main components: β-conglycinin and glycinin, which correspond to 7S and 11S globulins, respectively (Shewry et al. 1995). Both are contained inside vacuoles of seed cells and make up around 80% of storage proteins. β-Conglycinin is a trimeric protein consisting of three subunits α, α′, and β with approximately molecular weights of 67, 71, and 50 kDa, respectively (Utsumi 1992). Moreover, this study found that pure lectin (PNA) and its conjugates (PNA-CHNPs) show a single band with a molecular weight of 32 kDa. These results are similar to Sun et al. (2011), who showed that SDS-PAGE for peanuts lectins and the results identified a single band with a molecular weight of 29 kDa.

This study shows the absorbance spectra at wavelengths ranged between 200 and 400 nm for chitosan nanoparticles (CHNPs), pure lectins (PHA, PNA, and SBA), and their conjugates (PHA-CHNPs, PNA-CHNPs, and SBA-CHNPs), as shown in Fig. 2. The results indicate that the conjugation of chitosan nanoparticles with pure lectins resulted in a shift in the absorbance of chitosan 3.767 and pure lectins 3.503, 3.335, and 3.474 to be 0.624, 0.427, and 0.355 of the three conjugates, respectively. This shift was attributed to the shell thickness of the pure lectins surrounding the chitosan nanoparticles (CHNPs), indicating successful conjugation. These results align with Liu et al. (2010), who recorded a significant decrease in absorbance in the biotinylated chitosan solution compared to the chitosan solution.

In this work, the formed conjugates PHA-CHNPs, PNA-CHNPs, and SBA-CHNPs showed hemagglutination activity against all human blood types, suggesting the existence of active lectins in the conjugates. These results agree with the previous studies which demonstrated that phytohemagglutinin lectins (PHA), peanut lectins (PNA), and soybean agglutinin (SBA) can agglutinate all types of human red blood cells (Sharon and Lis 2004; Hamed et al. 2017).

The hemagglutination characteristic of lectins arises from their interactions with specific glycoproteins found on red blood cells to which lectins can attach (Moreira et al. 1991). Furthermore, lectins have a specific affinity for carbohydrates; for example, peanut agglutinin (PNA) specifically binds to the carbohydrate sequence Gal-β (1,3) GalNAc, while soybean agglutinin (SBA) is capable of binding N-acetyl-D-galactosamine and galactose (Takagi et al. 1988). Some lectins can identify and bind to complex glycan structures, such as PHA lectins. Their complex structures boost their binding strength and affinity for glycan ligands (Maliarik and Goldstein 1988). These findings can explain the positive hemagglutination results of three conjugates and provide a reason for the higher titer levels of hemagglutination (HA) observed in PHA-CHNPs in this study.

Nanoparticles penetrate the bacterial cell wall more effectively than antibiotics, leading to a larger inhibition zone (Abdullah et al. 2022). The present study recorded significant inhibition zones of chitosan nanoparticles using three different concentrations (2.5, 5, 10 and mg/ml) against five strains of bacteria tested. The largest inhibition zones for chitosan nanoparticles (10 mg/ml) were 24 ± 1 and 23.33 ± 1.53 mm against Gram-positive *Enterococcus faecalis* and Gram-negative *Salmonella typhimurium* respectively. These findings are stronger than those published by Alhazmi et al. (2022) who reported that the largest inhibition zone of chitosan nanoparticles (10 mg/ml) was 12.4 mm for *Enterococcus faecalis*, while the inhibition zone of chitosan nanoparticles (5 mg/ml) was 10 ± 1 and 12.67 ± 1.15 mm for *Escherichia coli* and *Staphylococcus aureus*, respectively. These findings were greater than those of Divya et al. (2017), who found that the inhibition zones of chitosan nanoparticles were 9.6 and 10.3 mm for *Escherichia coli* and *Staphylococcus aureus*, respectively.

Several factors can account for the differences in previous CHNP results. Smaller chitosan nanoparticles generally have a higher surface area-to-volume ratio, which can enhance their antibacterial activity by allowing for better interaction with bacterial membranes. The effectiveness of chitosan nanoparticles often depends on their concentration; higher concentrations may result in more pronounced antibacterial effects. Different bacterial strains may exhibit varying susceptibility to chitosan nanoparticles (Chandrasekaran et al. 2020).

The addition of nanoparticles to antibacterial agents increases their capacity to penetrate the bacterial cell and create a strong inhibition zone (Kim et al. 2007). Moreover, the structural difference between Gram-negative and Gram-positive bacteria results in varying antibacterial activity. The lectin finds it more difficult to penetrate the outer membrane and cell wall of Gram-negative bacteria and reach the periplasmic space. In contrast, Gram-positive bacteria exhibit a higher level of peptidoglycan, which offers more opportunities for interaction with the lectin (Silva and Araújo 2021).

The antibacterial activity of lectins is due to their ability to bind to the carbohydrate components of the bacterial cell wall or extracellular glycans. Antibacterial lectins can bind strongly to N-acetyl-D-acetylglucosamine (NAG), N-acetyl-D-muramic acid (NAM), and tetrapeptide components found in the cell walls of Gram-positive bacteria. They also interact with lipopolysaccharides (LPS) found in Gram-negative bacteria (Ayouba et al. 1994; Qadir et al. 2013; Lagarda-Diaz et al. 2017). In this study, PHA-CHNPs, PNA-CHNPs, and SBA-CHNPs show significant inhibition zones against all tested Gram-positive and Gram-negative bacteria. The largest inhibition zones were 55.67 ± 4.04 mm for PHA-CHNPs against *Enterococcus faecalis*, followed by 38.67 ± 5.51 and 36.3 ± 0.15 for PHA-CHNPs and PNA-CHNPs against *Salmonella typhimurium*, respectively. Moreover, the conjugate of PNA-CHNPs shows a higher inhibition zone (34 ± 2.65 mm) than 90% fractionated lectins extracted from the *Phaseolus vulgaris* seeds (15 mm) against *Staphylococcus aureus* (Hamed et al. 2017).

In this study, the MIC of chitosan nanoparticles (10 mg/ml) was 48, 12, and 48 µg/ml against *Escherichia coli*, *Salmonella typhimurium*, and *Staphylococcus aureus*, respectively. These results are different from the results by Qi et al. (2004), who recorded MIC values of chitosan nanoparticles 16, 4, and 4 µg/ml against *Escherichia coli*, *Salmonella typhimurium*, and *Staphylococcus aureus*, respectively. In addition, Divya et al. (2017) recorded that the MIC of chitosan nanoparticles (5 mg/ml) was 50 and 30 mg/ml against *Escherichia coli* and *Staphylococcus aureus*, respectively.

In this study, for the first time to our knowledge, MIC gave no inhibition for pure lectins (PHA, PNA, and SBA) against *Escherichia coli*, but their conjugates PHA-CHNPs, PNA-CHNPs, and SBA-CHNPs gave potent inhibition against same bacteria 48, 96, and 84 µg/ml, respectively. Moreover, PNA-CHNPs gave the lowest MIC value of 12 µg/ml against *Salmonella typhimurium*, and the lowest MIC values of PHA-CHNPs and SBA-CHNPs were 1.5 and 12 µg/ml, respectively, against *Enterococcus faecalis*. Whereas prior to conjugation, the MICs for chitosan nanoparticles (CHNPs) against *Enterococcus faecalis* were reduced to 24 µg/ml. These findings are consistent with Rubeena et al. (2020), who reported that MIC of shrimp lectin (Md-Lec) decreased from 12.5 to 3.13 µg/ml after conjugation with pectin-capped copper sulfide nanoparticles (pCuS NPs) against *Enterococcus faecalis*.

For the first time, this research shows microphotographs of chitosan nanoparticles (CHNPs), pure lectins (PHA, PNA, and SBA), and their conjugates (PHA-CHNPs, PNA-CHNPs, and SBA-CHNPs). The microphotographs clearly showed a unique pattern of increase in conjugate size and significant binding between lectins and chitosan nanoparticles. These findings agree with Mishra et al. (2014) who confirmed that the increase in conjugate size proves the effective interaction between lectin and chitosan nanoparticles.

As far as we know, this study is the first to report significant cytotoxic results in terms of cell viability percentages and IC_50_ values for pure lectins (PHA) and two conjugates with chitosan nanoparticles (PHA-CHNPs and PNA-CHNPs) against three cancer cell lines: colorectal cancer (HT-29), human melanoma (A375), and tongue carcinoma (HNO-97). Moreover, the results of chitosan nanoparticles (CHNPs), pure lectins (PHA, PNA, and SBA), and their conjugates (PHA-CHNPs, PNA-CHNPs, and SBA-CHNPs) are not cytotoxic to two normal cell lines: oral epithelial cell (OEC) and human skin fibroblast (HSF). The selective cytotoxicity of conjugates against cancer cells compared to normal cells is due to cancer cells’ ability to change glycan structures on their surfaces, as well as their ability to grow and metastasize. Furthermore, the ability of lectins to bind with carbohydrates found on cancer cells could affect expression profiles, metastatic distribution patterns, and lymphatic invasion prognosis (Konno et al. 2002).

Phytohemagglutinin (PHA) is considered a complex lectin that consists of PHA-L and PHA-E, each with distinct carbohydrate-binding specificities and properties. PHA exhibits a complex binding profile, primarily recognizing N-acetyl-D-glucosamine and D-mannose, but it can also interact with other glycan structures. Thus, PHA can induce many biological processes, including antibacterial activity, cell agglutination, and anticancer activity (Wang et al. 2021).

In this study, PHA-CHNPs at a concentration of 100 μg/ml show the highest cytotoxic activity against all cancer cell lines, and the most sensitive cancer cell lines were tongue carcinoma (HNO-97) with reduction of cell viability by 38.78 ± 1.85% and IC_50_ 31.69 μg/ml, while using pure PHA lectin gave 91.92 ± 1.32% and IC_50_ more than 100 μg/ml. The next sensitive cancer cell line using PHA-CHNPs at a concentration of 100 μg/ml was colorectal cancer (HT-29) with a cell viability percentage of 49.88 ± 1.11 and IC_50_ value of 88.68 μg/ml, while pure PHA lectins have a low cytotoxic effect against colorectal cancer (HT-29) with a cell viability percentage of 57.93 ± 1.64% and IC_50_ 128.53 μg/ml. The lowest response was recorded in A375 (human melanoma) treated with 100 μg/ml of PHA-CHNPs with viability of 66.92 ± 3.60% and IC_50_ value of 135.58 μg/ml. These results were supported by a previous study indicating that the anticancer effect of PAC-CHNPs (proanthocyanidin chitosan nanoparticles) was higher than proanthocyanidin (PAC) on HT-29 cells. The PAC-CHNPs showed significant cytotoxic effects on HT-29 cells, causing the death of several cancer cells at a low concentration at 6.25 μg/ml (50.83 ± 0.82), while PAC maintained cell viability over 60% at the same concentration (Mani et al. 2020).

To the best of our insight, for the first time, this study presented microphotographs of cancer cell lines treated with chitosan nanoparticles (CHNPs), pure lectins (PHA, PNA, and SBA), and their conjugates (PHA-CHNPs, PNA-CHNPs, and SBA-CHNPs). The photomicrographs show distinct morphological differences compared to the control cancer cell lines. The study showed that PHA-CHNPs at a concentration of 100 μg/ml induced apoptosis in three cancer cell lines, as shown by cell shrinkage, decreasing the intercellular junctions, and proliferation rates. These results aligned with a previous study showing photomicrographs of PAC-CHNPs leading to a significant decrease in tumor cell proliferation (HT-29), characterized by cell shrinkage, nuclear shrinkage, cytoplasmic shrinkage, and nuclear condensation (Mani et al. 2020).

This study suggests that both pure PHA and the conjugate PHA-CHNPs have potent anticancer properties, with high-efficiency cytotoxicity against tongue carcinoma (HNO-97), colorectal cancer (HT-29), and human melanoma (A375). These findings agree that the carbohydrate-binding specificity of lectins makes them essential tools for studying cancer cell carbohydrate expression profiles, metastatic distribution patterns, and cancer treatment (Konno et al. 2002). Additionally, chitosan nanoparticles (CHNPs) have a vital role as secure carriers and anticancer drugs (Ponraj et al. 2017).

In conclusion, this study synthesized novel active biomaterials based on the conjugation of chitosan nanoparticles (CHNPs) with pure plant lectins (PHA, SBA, and PNA). The conjugation was performed using a successful two-step method using glutaraldehyde as an effective crosslinking agent. SDS-PAGE profile shows almost the same pattern and molecular weight for pure lectins and lectin-conjugated chitosan nanoparticles. TEM microphotographs show different structures of the conjugates compared to pure lectins and chitosan nanoparticles. Moreover, this study recorded that all lectin-conjugated chitosan nanoparticles have antibacterial effects against tested Gram-negative and Gram-positive bacteria. All conjugates are safe for oral epithelial cells (OEC) and human skin fibroblasts (HSF), suggesting their potential application in antibacterial skincare products (ointments, creams, and sprays) and oral hygiene products such as toothpaste and mouthwash. However, some of them exhibit cytotoxic effects on tongue carcinoma (HNO-97), colorectal cancer (HT-29), and human melanoma (A375). These findings suggest that plant lectin–conjugated chitosan nanoparticles are promising bioactive materials for combating bacterial resistance and controlling hazardous infections. Furthermore, their anticancer properties make them the future tools in further studies for therapeutic cancer diseases. This work recommends doing more research in this field to explore the benefits of plant lectin–conjugated nanoparticles for future applications in the medical industry, drug delivery, and cancer treatment.

## Supplementary Information

Below is the link to the electronic supplementary material.

Supplementary file1 (PDF 3615 KB)

## Data Availability

Data are available upon request.
