# Supplementary material for: Antibacterial and anticancer activities of three novel lectin-conjugated chitosan nanoparticles
Source: Appl Microbiol Biotechnol. 2024 Nov 27;108:524. doi: 10.1007/s00253-024-13344-7 (PMC11602803; doi:10.1007/s00253-024-13344-7)
Supplement: Supplementary file 1 [file 253_2024_13344_MOESM1_ESM.pdf]

Applied Microbiology and Biotechnology

Antibacterial and anticancer activities of three novel lectin conjugated chitosan nanoparticles.

***Mervat Mounir Soliman[1], Einas Hamed El-Shatoury[1]**
Magda Mahmoud Ibrahim El-Araby[2]

[1]Department of Microbiology, Faculty of Science, Ain-Shams University
[2] Department of Botany, Faculty of Science, Ain-Shams University
* Corresponding author: Emails: mervatmonir_p@sci.edu.eg or
mervatmounir84@yahoo.com, Telephone: 00201141175244

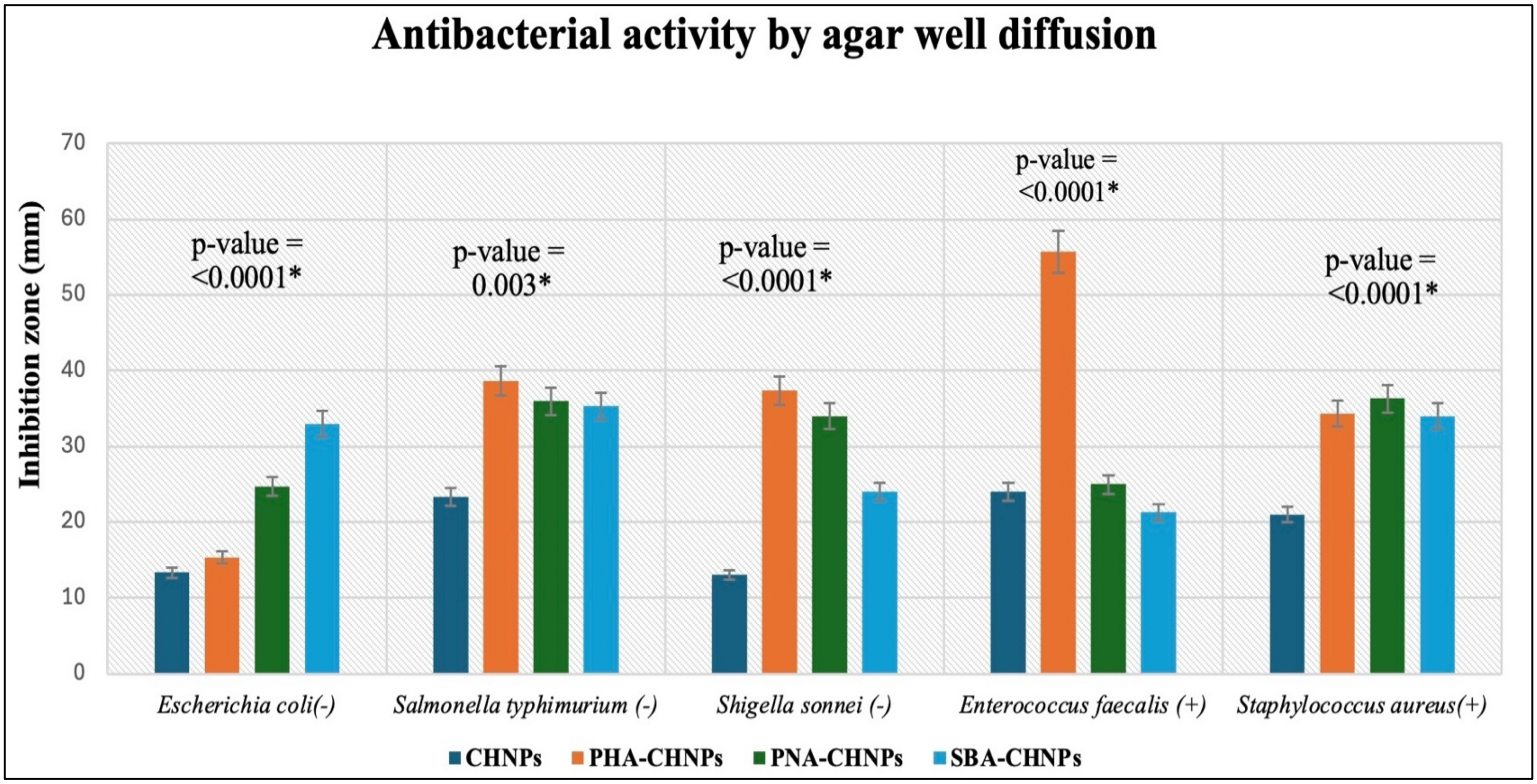

Figure S1 shows the antibacterial activity of chitosan nanoparticles (CHNPs) of concentration 10 mg/ml and three lectins conjugated chitosan nanoparticles (PHA-CHNPs, SBA-CHNPs, and PNA-CHNPs) using 400 µl of glutaraldehyde (%25) against five tested strains Gram-negative bacteria (-) and Gram-positive bacteria. Statistically significant results were observed in all cases, as indicated by the p-values ($p < 0.05$).

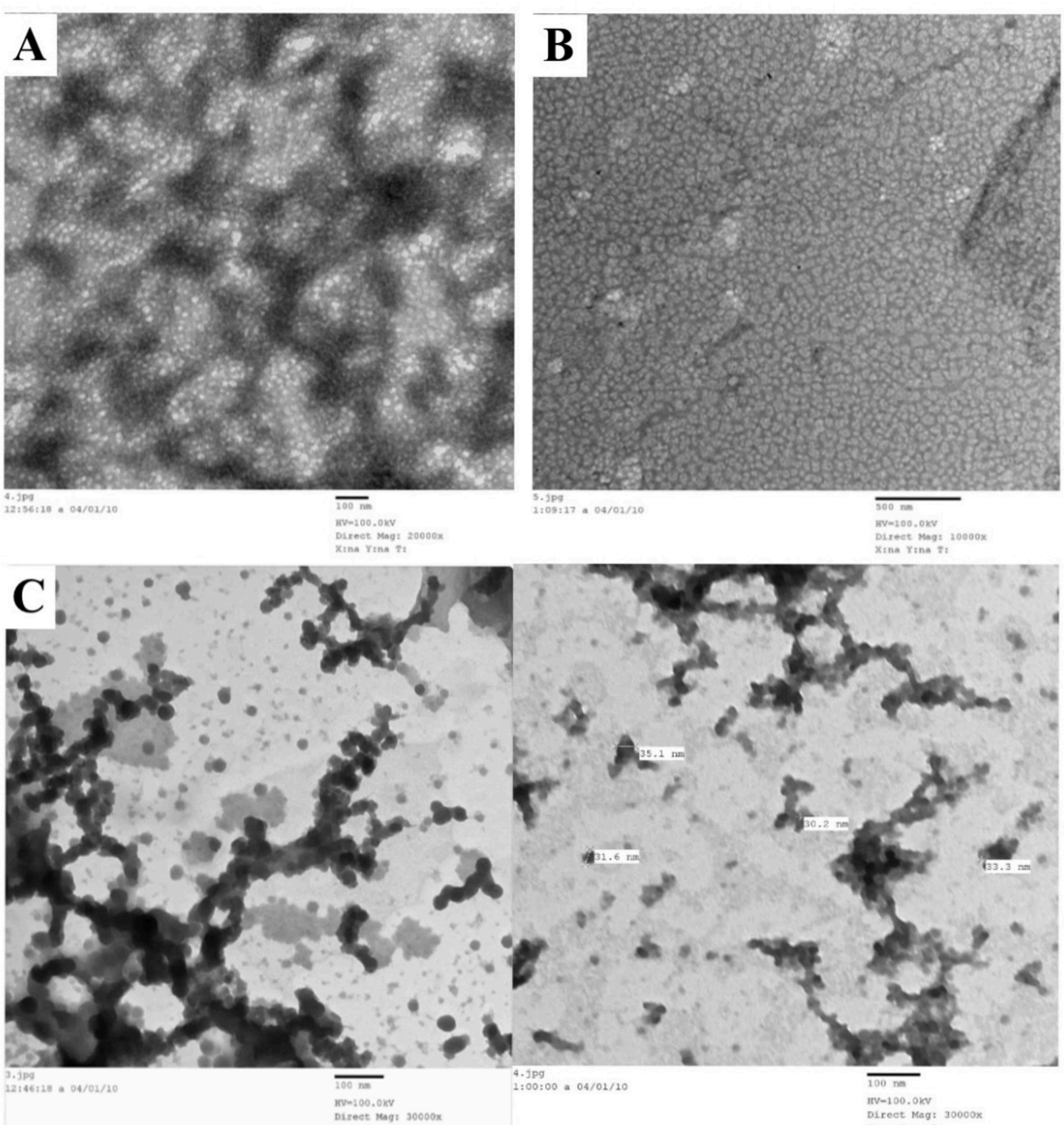

Figure S2 shows TEM photomicrographs of **A** = chitosan nanoparticles; **B=** pure lectins (PHA); and **C** = lectin conjugated chitosan nanoparticles **(PHA-CHNPs)** using 400 µl of 0.25% glutaraldehyde. PHA = phytohaemagglutinin lectin.

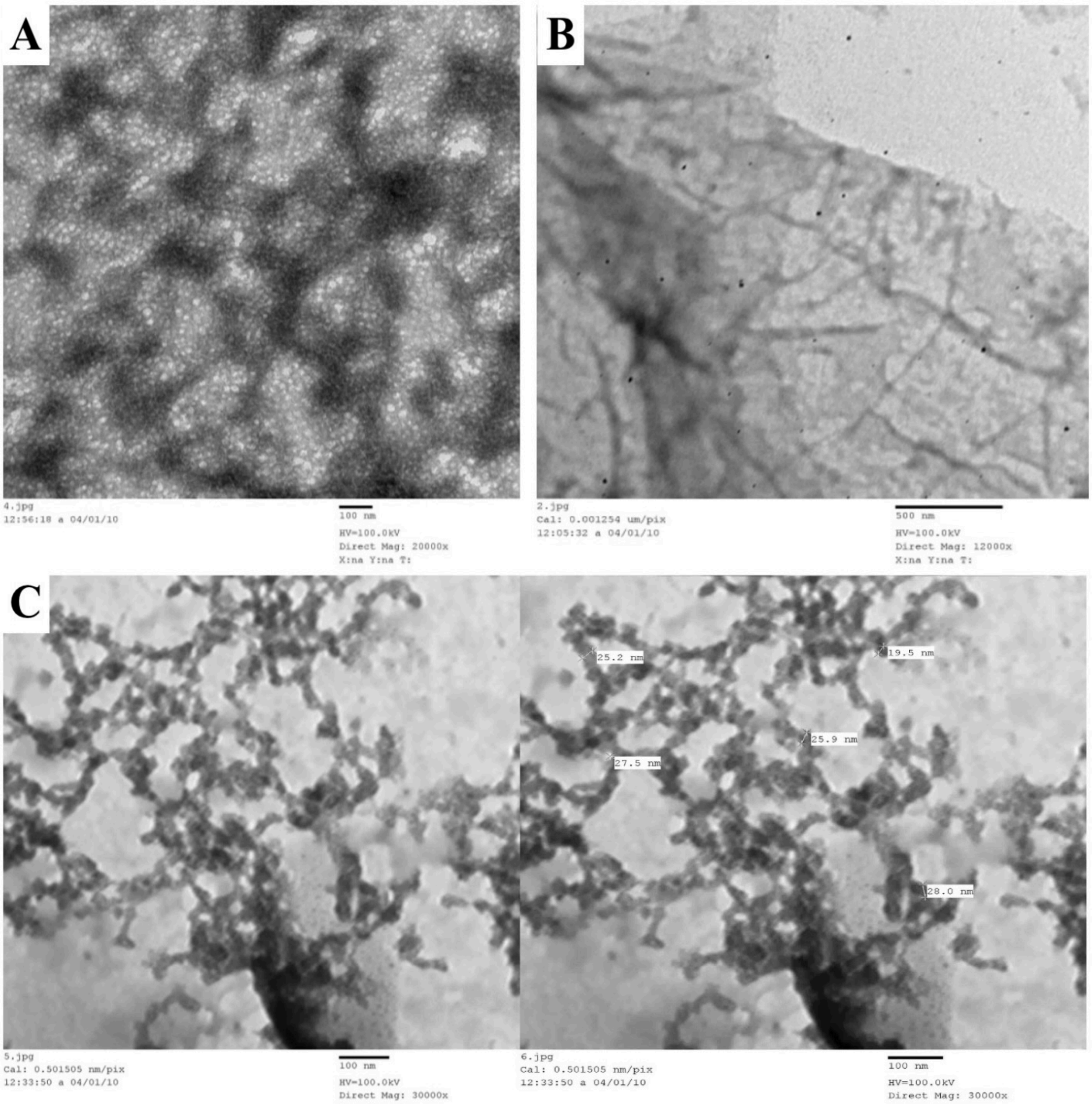

Figure S3 shows TEM photomicrographs of **A =** chitosan nanoparticles; **B=** pure lectins (PHA); and **C =** lectin conjugated chitosan nanoparticles **(PNA-CHNPs)** using 400 µl of 0.25% glutaraldehyde. PNA = peanut agglutinin lectin.

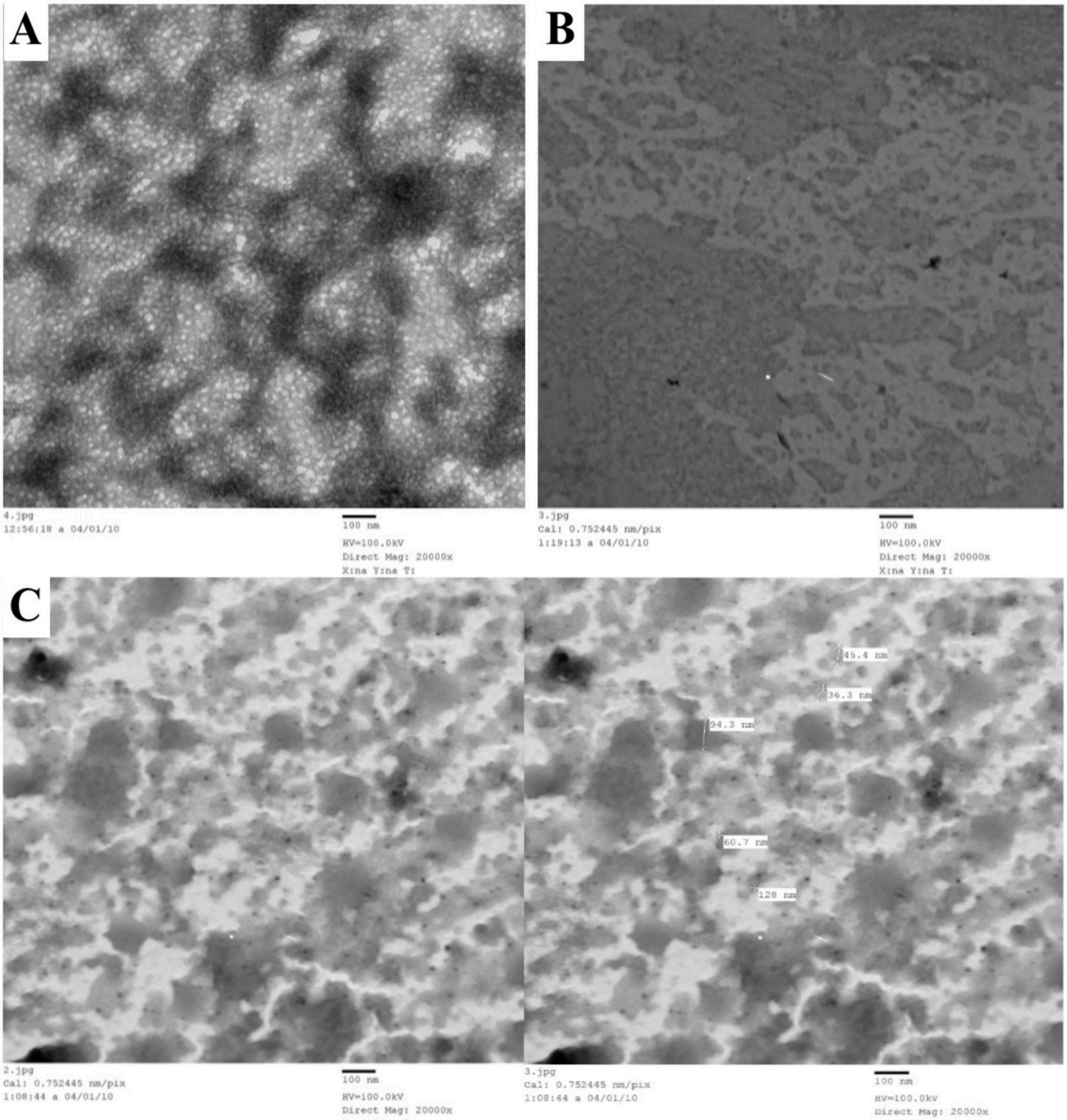

Figure S4 shows TEM photomicrographs of **A =** chitosan nanoparticles; **B=** pure lectins (PHA); and **C=** lectin conjugated chitosan nanoparticles **(SBA-CHNPs)** using 400 µl of 0.25% glutaraldehyde. SBA = soybean agglutinin lectin.

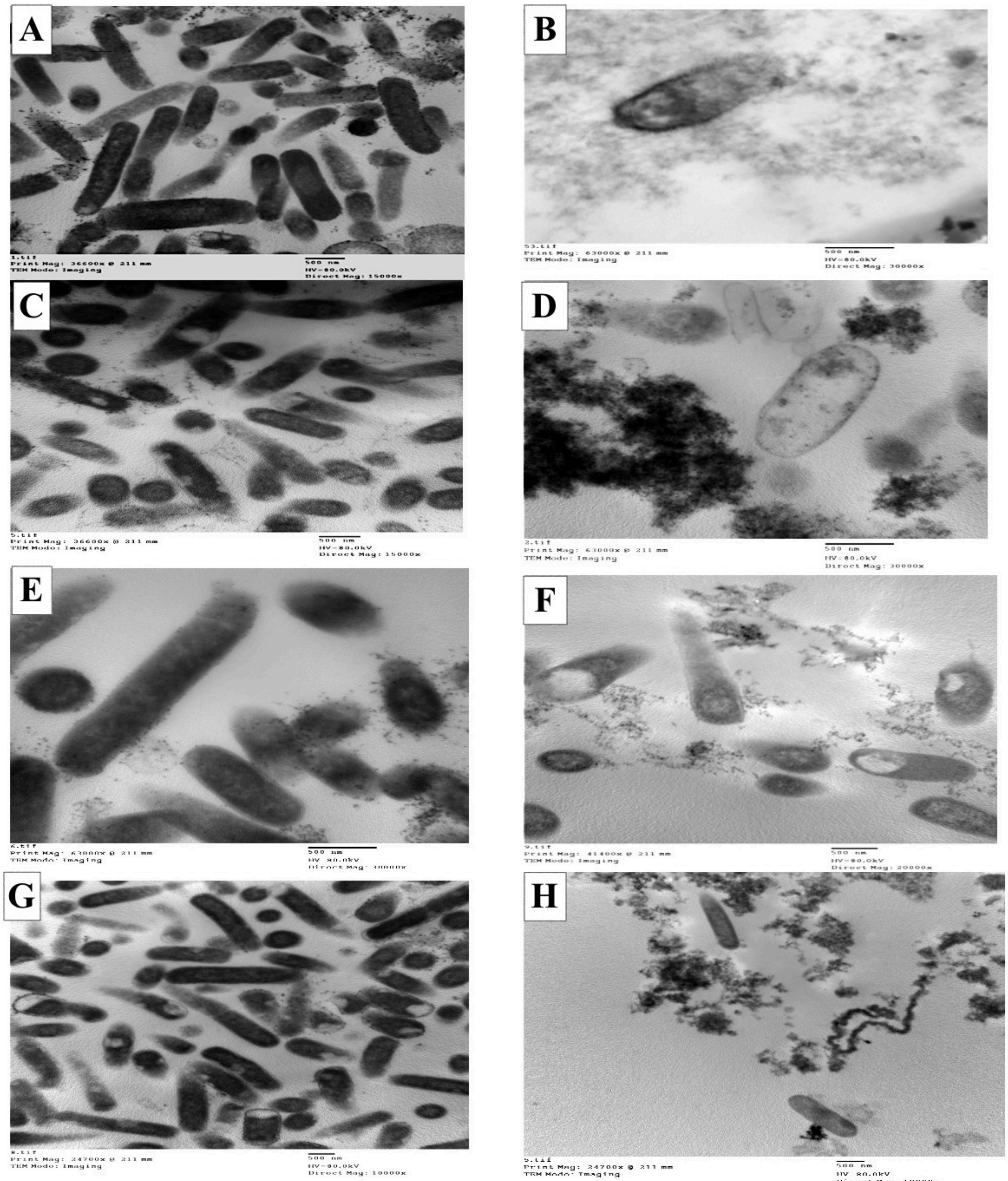

Figure S5 shows TEM photomicrographs of ***Escherichia coli,*** **A=** control without treatmeant , **B=**chitosan nanoparticles; **C, E, G=** pure lectins PHA, PNA, and SBA respectively; **D, F, H=**lectins conjugated chitosan nanoparticles PHA-CHNPs, PNA-CHNPs, and SBA-CHNPs respectively using 400 µl of 0.25% glutaraldehyde. PHA = Phytohaemagglutinin lectin, PNA= Peanut agglutinin lectin, and SBA=Soybean agglutinin lectin.

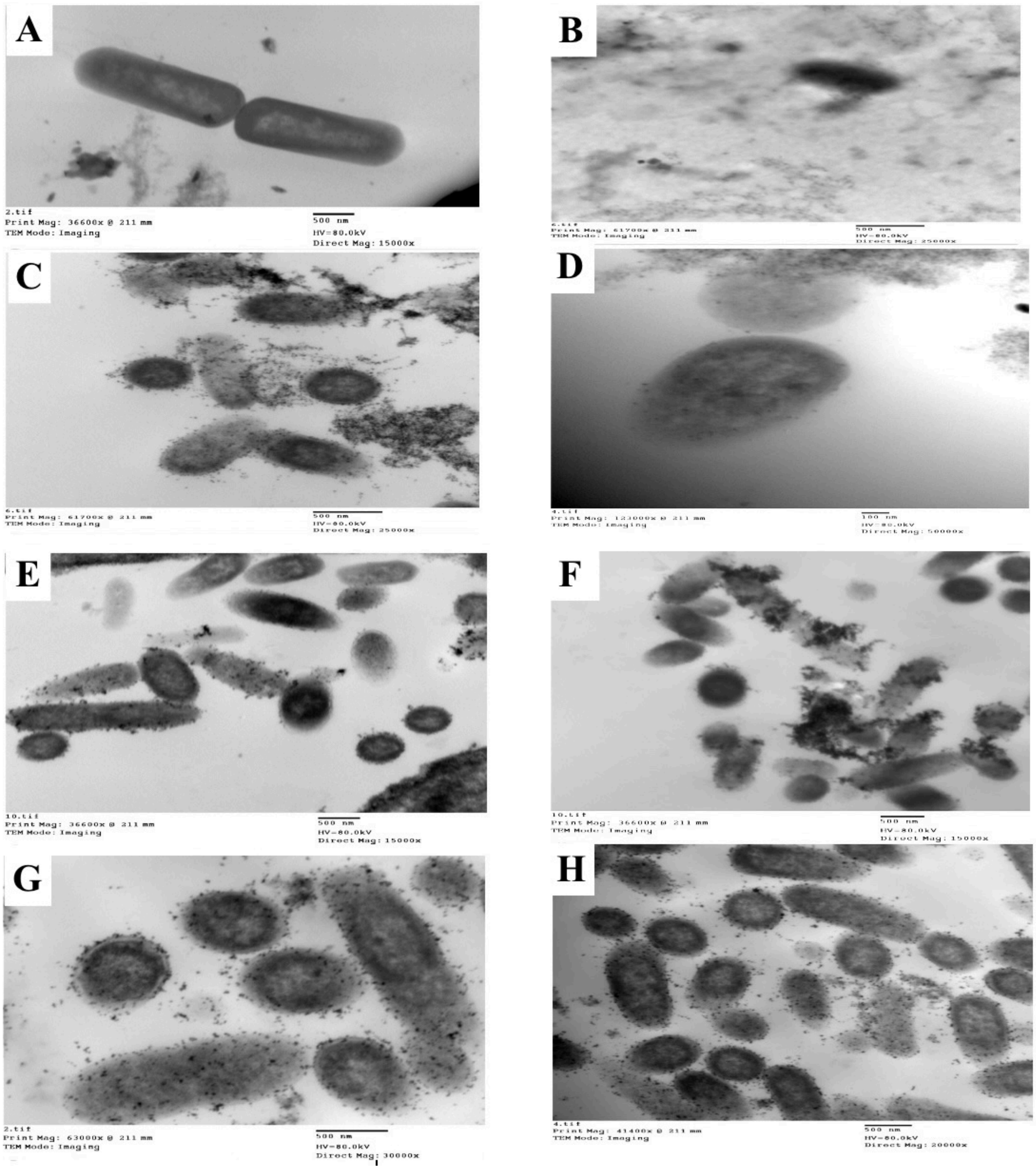

Figure S6 shows TEM photomicrographs of ***Salmonella Typhimurium,*** **A=** control without treatmeant , **B=**chitosan nanoparticles; **C, E, G =** pure lectins PHA, PNA, and SBA respectively; **D, F, H =**lectins conjugated chitosan nanoparticles PHA-CHNPs, PNA-CHNPs, and SBA-CHNPs respectively using 400 µl of 0.25% glutaraldehyde. PHA = phytohaemagglutinin lectin, PNA= peanut agglutinin lectin, and SBA=soybean agglutinin lectin.

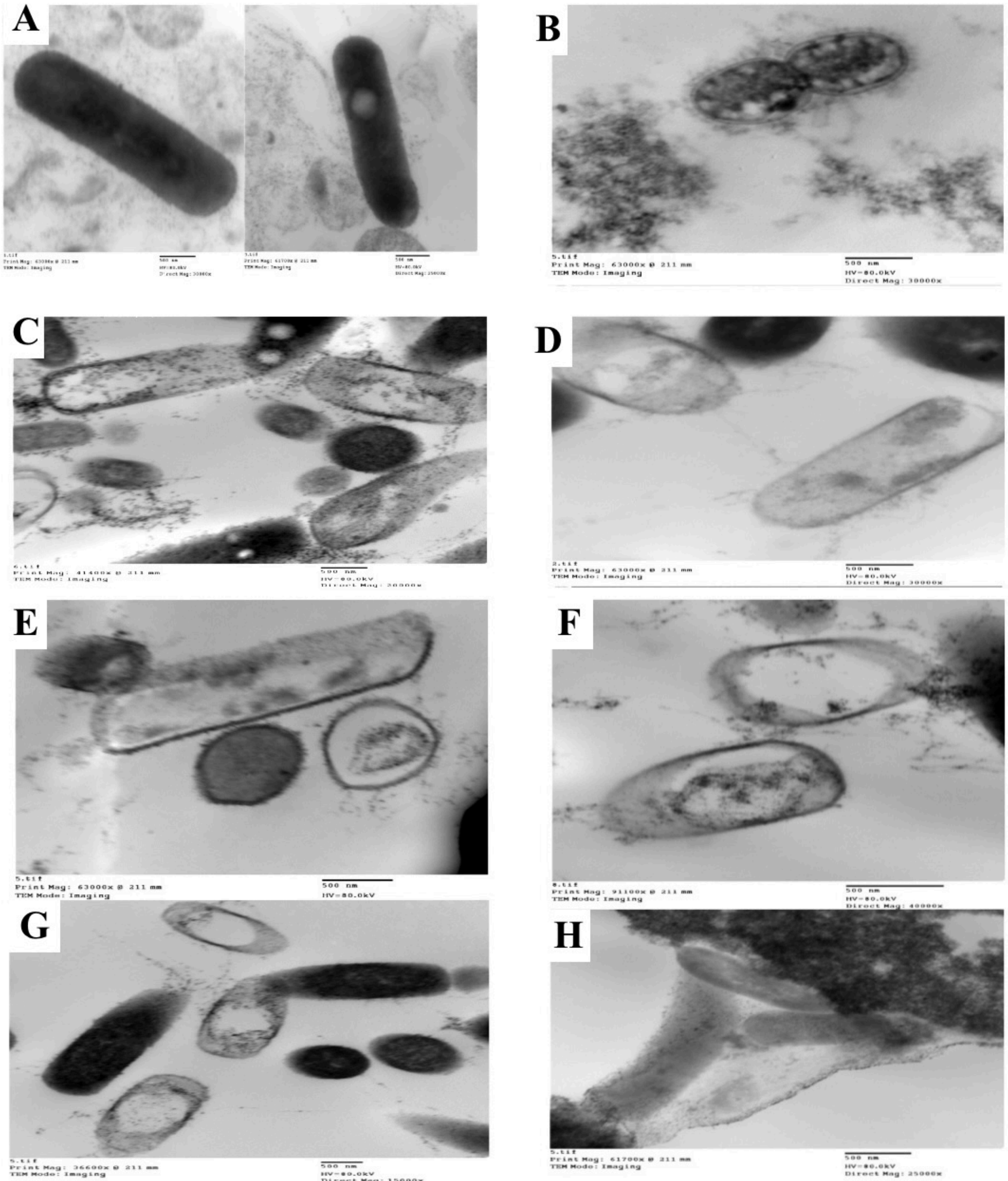

Figure S7 shows TEM photomicrographs of ***Shigella Sonnei,*** **A=** control without treatmeant , **B=**chitosan nanoparticles; **C, E, G =** pure lectins PHA, PNA, and SBA respectively; **D, F, H** =lectins conjugated chitosan nanoparticles PHA-CHNPs, PNA-CHNPs, and SBA-CHNPs respectively using 400 µl of 0.25% glutaraldehyde. PHA = phytohaemagglutinin lectin, PNA= peanut agglutinin lectin, and SBA=soybean agglutinin lectin.

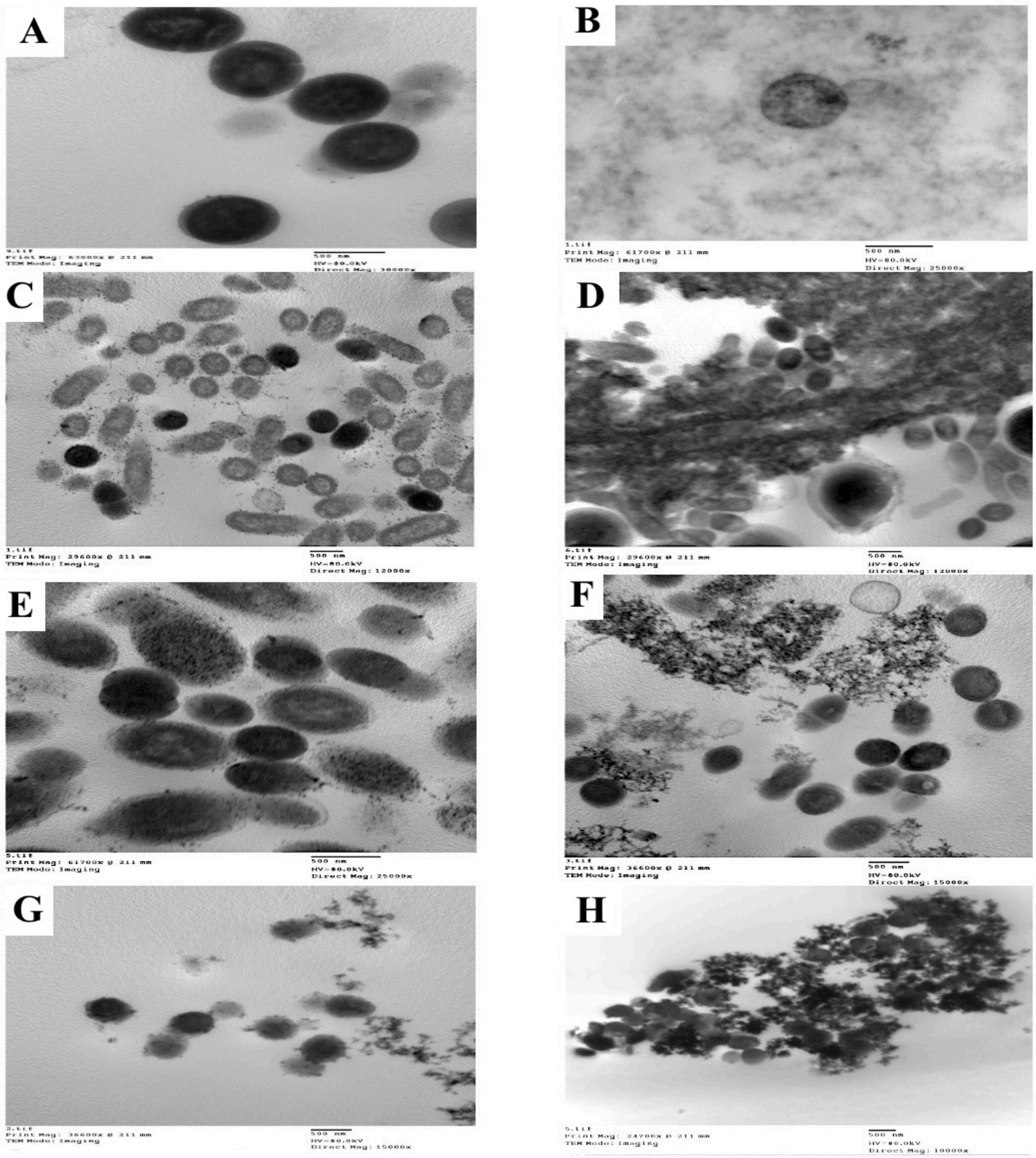

Figure S8 shows TEM photomicrographs of ***Enterococcus faecalis,*** **A=** control without treatmeant , **B=**chitosan nanoparticles **C, E, G =** pure lectins PHA, PNA, and SBA respectively; **D, F, H** =lectins conjugated chitosan nanoparticles PHA-CHNPs, PNA-CHNPs, and SBA-CHNPs respectively using 400 µl of 0.25% glutaraldehyde. PHA = phytohaemagglutinin lectin, PNA= peanut agglutinin lectin, and SBA=soybean agglutinin lectin.

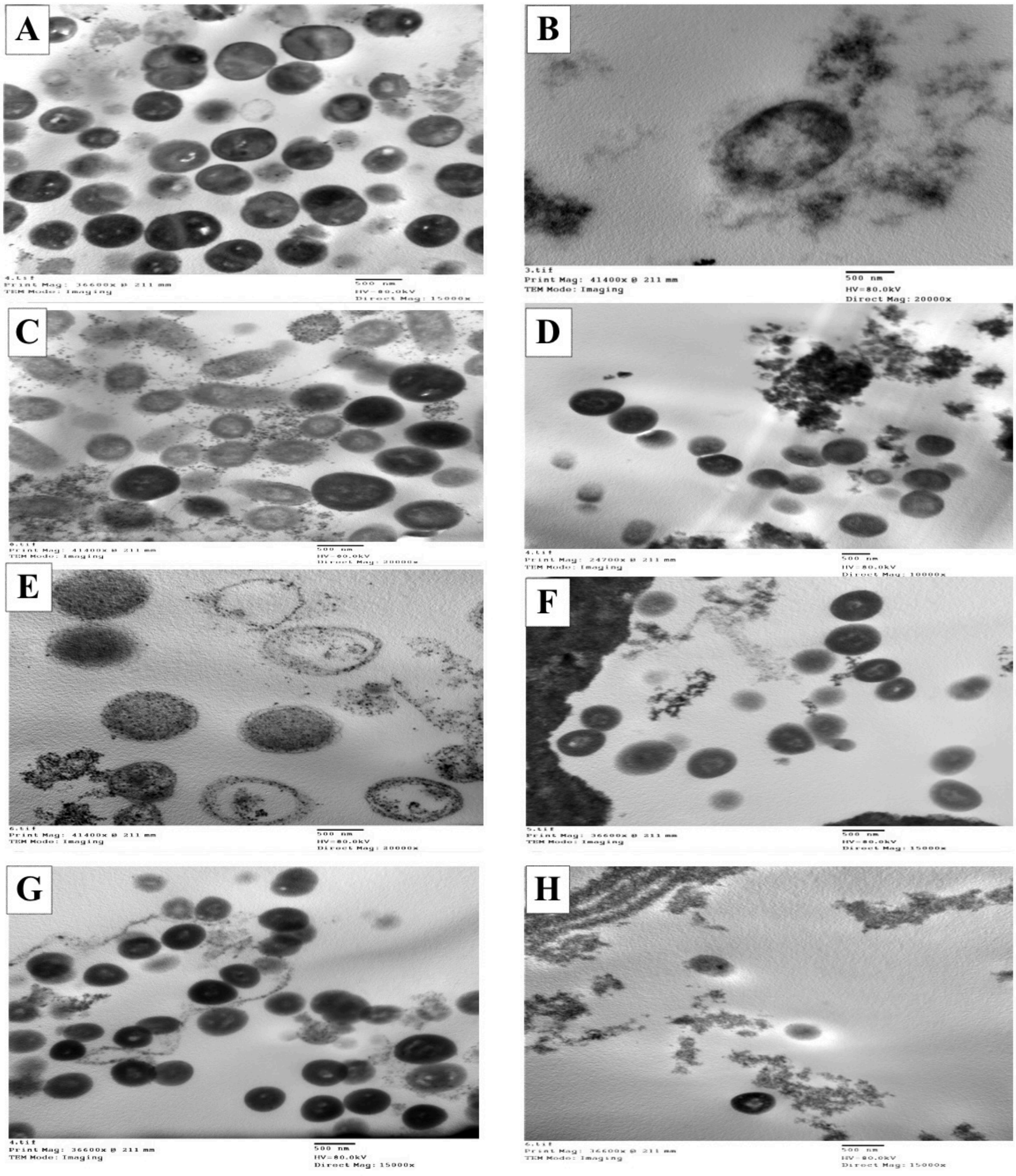

Figure S9 shows TEM photomicrographs of ***Staphylococcus aureus***, **A=** control without treatmeant , **B=**chitosan nanoparticles; **C, E, G =** pure lectins PHA, PNA, and SBA respectively; **D, F, H** =lectins conjugated chitosan nanoparticles PHA-CHNPs, PNA-CHNPs, and SBA-CHNPs respectively using 400 µl of 0.25% glutaraldehyde. PHA = phytohaemagglutinin lectin, PNA= peanut agglutinin lectin, and SBA=soybean agglutinin lectin.

Table S1: Protein concentration of three lectins conjugated chitosan nanoparticles (PHA-CHNPs, PNA-CHNPs, and SBA-CHNPs) using three volumes (200, 300, and 400) of 0.25% glutaraldehyde after estimation by nanodrop technique.

Conjugates	Volumes of 0.25% glutaraldehyde	Concentration (mg/ml)
PHA -CHNPs	200	0.066
	300	0.146
	400	0.432
PNA -CHNPs	200	0.056
	300	0.181
	400	0.629
SBA- CHNPs	200	0.111
	300	0.254
	400	0.960

PHA-CHNPs = Phytohemagglutinin lectin conjugated chitosan nanoparticles
PNA-CHNPs = Peanut agglutinin lectin conjugated chitosan nanoparticles
SBA-CHNPs =Soybean agglutinin lectin conjugated chitosan nanoparticles

Table S2: Haemagglutination activity (HA) of three lectins conjugated chitosan nanoparticles (PHA-CHNPs, PNA-CHNPs, and SBA-CHNPs) using 400 µl of 0.25% glutaraldehyde against human blood groups A, B, AB, and O

.

Conjugates	Human blood groups			
	A	B	AB	O
	Haemagglutination Activity (titer)			
PHA -CHNPs	256	128	128	256
PNA- CHNPs	8	8	16	8
SBA- CHNPs	32	32	64	16

PHA-CHNPs = Phytohemagglutinin lectin conjugated chitosan nanoparticles
PNA-CHNPs = Peanut agglutinin lectin conjugated chitosan nanoparticles
SBA-CHNPs =Soybean agglutinin lectin conjugated chitosan nanoparticles
Titer = The reciprocal of the heights dilution of protein solution showing visible hemagglutination.
